# Miscellaneous

**Published:** 2008-10

**496**

**Prevalence and pattern of complementary and alternative medicine use among cancer patients: A questionnaire based study**

Joshi D^1^, Gupta P^1^, Doval DC^2^, Gupta YK^1^, Talwarl V^2^, Bakshi S^1^

**^1^All India Institute of Medical Sciences, New Delhi, India, ^2^Rajiv Gandhi Institute of Health Sciences, New Delhi, India.**

**Background:**

Cancer patients are known to use Complementary and Alternative Medicine (CAM). However its extent and its influencing factors have not been documented in India. This study was conducted to assess the percentage of patients who used other forms of treatment in addition to or instead of conventional therapy and the pattern of CAM use.

**Materials and Methods:**

A questionnaire based study was conducted among patients seeking treatment for cancer at two tertiary care centres at New Delhi. Information was gathered under the following headings i) Demographics ii) Type of cancer, stage, conventional cancer treatment and its side effects iii) CAM use for cancer, reasons for the same, beliefs regarding CAM and its side effects.

**Results:**

A total of 101 patients (19 – 79 years) were interviewed. 71.3% of the patients were male and 29.7% were female. 87.1% lived in urban areas and 12.9% in rural areas. 27.7% of the participants used CAM of which 67.9% used Ayurveda, 21.4% used Yoga, 17.9% used Homeopathy and 25% used other forms of CAM. 78.6% of patients used CAM after having used conventional medicine of which 31.8% added CAM to conventional therapy in an effort to try anything that could help, 22.7% started CAM since they were disappointed that conventional treatment was not working and 40.1% said there was no specific reason. 46.4% of those who used CAM felt it helped them, 42.9% felt it didn’t help and 10.7% couldn’t say. 39.3% of those who used CAM came to know about it from other patients, 35.7% heard it from family and/ or friends and the rest from other sources; however majority used it under the guidance of a CAM practitioner.

**Conclusion:**

CAM use is a common phenomenon in the cancer patients. However, patients. experience with CAM has yielded mixed results on whether CAM is beneficial or not. The effect of the use of alternative forms of medicine needs to be investigated in randomized clinical trials for efficacy and safety as an adjunct to conventional medicine for management of cancer.

**497**

**Effect of roots of *Rubia cordifolia* in streptozotocinnicotinamide induced type-II diabetes mellitus**

Somani RS^1^, Singhai AK^2^

**^1^Sinhgad College of Pharmacy, Pune, India; ^2^Dept. of Pharm. Sci., Dr. HS Gour University, Sagar, India.**

**Objective:**

The objective of this study was to ascertain the scientific basis for the use of *Rubia cordifolia* in the management of diabetes using streptozotocin-nicotinamide induced type-II diabetic rats.

**Methods:**

Alcoholic extract of *Rubia cordifolia* (RCAE) was prepared by maceration. Ethyl acetate fraction (RCEAF) was obtained from RCAE by column chromatography. Streptozotocin-nicotinamide was used to induce type-II diabetes mellitus. The various parameters studied includes blood glucose levels, serum lipid levels, insulin and glycosylated hemoglobin, liver and skeletal muscle glycogen content, and histopathology of pancreas. RCEAF (200 mg/kg, p.o.) was administered for four weeks in normal fasted and STZ- nicotinamide - induced diabetic rats. The blood glucose levels were estimated by glucose oxidase- peroxidase reactive strips and other biochemical parameters were estimated using diagnostic kits (Biolabs, Mumbai).

**Result:**

Repeated dose treatment for four weeks of RCEAF caused significant (*P*< 0.05) decrease in blood glucose, serum total cholesterol, triglyceride, glycosylated hemoglobin and increase in serum HDL- cholesterol and insulin as compared to diabetic control rats. STZ- nicotinamide significantly (*P*< 0.05) caused sever depletion of liver and skeletal muscle glycogen content and pancreatic islet cells architecture showed degenerative changes. The administration of RCEAF for four weeks restored liver and skeletal muscle glycogen significantly (*P*< 0.05) and showed proliferation of islet cells.

**Conclusion:**

The present investigation shows that *Rubia cordifolia* has antidiabetic activity in STZ-nicotinamide induced type-II diabetic rats. However, further studies on insulin receptor and its mode of action will provide deeper insights for the discovery of better and safer therapeutics.

**498**

**Prevention and control of schistosomiasis mansoni after administration of artemether in experimentally infected mice**

Mahmoud M^1^, Abed EL Hamid S, Bedeer B, Mahboub M, El Nawasra N

**^1^Theodor Bilharz Reasearch Institute, Giza, Egypt; Faculty of Medicine for Girls, Al-Azhar University, Cairo, Egypt.**

The aim of this work is to study the antischistosomal effect of artemether injection (ART) on the prevention and control of *S. mansoni* either alone or combined with oral praziquantel (PZQ). In this work, 48 mice were infected with *S. mansoni* cercariae and divided into 4 groups. First group (infected control) received the vehicles of the drugs, the second received ART in 3 doses at 2, 3 and 6 weeks post infection each at 300mg/kg intramuscularly, third group received PZQ orally in a full curative dose (2x500mg/kg) at 6 weeks post infection, the last group received combined therapy with the same regimen of the previous 2 groups. Animals were sacrificed at 8 weeks post infection and parasitology parameters (worm count, distribution, % hepatic shift, uncoupling, oogram pattern, ova count), biochemical parameters (ALT, GGT, MDA, % viability of isolated hepatocytes) and histopathological parameters (granuloma diameters, granuloma type). Treatment with ART reduced the total number of worms by 95.7% with complete absence of all egg developmental stages and consequently no granulomatous lesions were formed. There was a significant reduction in the serum level of alanine aminotransferase (ALT) and gamma glutamyl transferase (GGT) and the level malondialdehyde (MDA) in liver homogenate compared to infected control group. Also, there was an improvement in the % viability of isolated hepatocytes combined therapy of ART and PZQ improve the activity of each drug alone.

**499**

**Low dose of statin in inhibition of chemical rat mammary carcinogenesis: A molecular approach**

Ghosh B^1^, Chatterjee M^1^, Mondal S^2^

**^1^Jadavpur University, India, ^2^WBUHS, India.**

Cardio-vascular diseases and Cancer are two leading diseases in the world. Statins act by blocking the conversion of 3-hydroxy-3- methylglutaryl coenzyme A (HMG CoA) to mevalonate, the precursor of cholesterol and effective drug in the prevention of cardiovascular diseases. Recent studies suggest that statins have additional effects independent of LDL cholesterol lowering. Abnormal angiogenesis; pro-angiogenic factors; few interleukins and cytokines; are common in both the conditions. In the experiment, the entire animals are divided into six groups to know the effect properly. Rats were randomized to receive atrovastatin or vehicle by gavage. Carcinogentreated groups that should receive a single tail vein injection of 7, 12 Dimethylbenz (α) anthracene (DMBA) at a dose of 0.5mg/100g weight in an oil emulsion at 50days of age. All the treatment will be withdrawn after week 31 and the rats are sacrificed. Histological evaluation of mammary tissue, Cell proliferation assay by BrdUcoupled Immunohistochemistry, Detection of apoptosis in situ, Immunohistochemical detection of Bax, Bcl2 and p53 proteins in situ were done. Data generated from this study would be analyzed by Standard Statistical Software mainly SPSS. Result showing statin modulates gene expression, influence apoptosis and cell growth. In conclusion, Statins is elucidated at molecular level; may have the beginning of a new chemo-preventive program.

**500**

**Drug development clues from scorpion venom against experimental osteoporosis**

Gomes A

**University of Calcutta, 92, APC Road, Calcutta, India.**

**Introduction:**

Osteoporosis (silent disease), is a worldwide socio-medical problem of the senior citizen especially the elderly woman. Therapeutic management available (estrogen, calcium and D3, alendronate, raloxifene, etc) has its own limitations (cost, ineffectiveness, side effects, etc). Several alternative therapies are mentioned in the folk and traditional medicine of China and India. The present communication is an effort to establish the antiosteoporosis activity of scorpion venom / toxin in experimental animal.

**Methods:**

Osteoporosis was developed in female albino rats by bilateral ovariectomy and methyl prednisolone and confirmed through urinary markers. A protein toxin (HBF1) was purified from Scorpion venom through ion exchange chromatography and its the antiosteoporosis activity was established through the urinary, serum and bone markers, and compared with osteoporosis rats.

**Results:**

A high MW protein toxin (HBF1) purified from the Indian black scorpion (*Heterometrus bengalensis*) venom through DEAE cellulose IEC followed by HPLC. The MW of the HBF1 was found to be 76.8 KDa and the first 20 amino acid sequence of HBF1 was found to be G-P-L-T-I-L-H-I-N-D-V-H-A-A/R-F-E-Q/G-F/G-N-T. HBF1 exhibited significant antiosteoporosis activity in Wister female albino rats, by restoring urinary (Ca, PO_4_, CRE, OH-P), serum/plasma (Ca, PO_4_, ALP, TRAP, PTH, T_3_, TSH, calcitonin osteocalcin, IL1, IL6 and TNFα) and bone minerals (Ca, P, Mg, Zn, Na) parameters, as compared with osteoporosis rats. The bone dimensions, morphology and histological changes observed in osteoporosis rats were restored in HBF1 treated rats.

**Conclusion:**

HBF1 is the first protein toxin isolated from scorpion venom exhibited antiosteoporosis activity. Further works at molecular level are in progress.

**501**

**Protective effect of aminoguanidine in diabetes induced hyperalgesia and vascular dysfunction**

Muhammad Afzal^1^, Patel NR^2^, Kanzariya NR^1^, Tripathi P^1^, Patel Rameshvar K^1^, Patel Natvar J^1^

**^1^S.K. Patel College of Pharmaceutical Education and Research, Kherva, India, ^2^Kalol Institute of Pharmacy, Kalol, Gujarat, India.**

**Introduction:**

Insulin-dependent diabetes mellitus (type-1 diabetes) is an inflammatory autoimmune disease associated with vascular permeability changes leading to many complications including nephropathy, retinopathy, hypertension, hyperalgesia, neuropathy and vascular dysfunction. Prolong and severe hyperglycemia in rats develops hyperalgesia leading to decrease in pain threshold, which increases pain sensitivity. Protective effect of aminoguanidine in diabetes induced hyperalgesia and vascular dysfunction were elucidated in the present study.

**Methods:**

In present study diabetes was induced by administration of 60 mg/ kg streptozotocin I.P. Aminoguanidine treatment was given for 4 weeks to diabetic animals. Hyperalgesic reaction were measured using Eddy’s hot plate method and radiant heat analgesiometer, while vascular dysfunction were accessed by relaxation response of Ach in phenylephrine induced precontractile rat thoracic aorta.

**Results:**

In Eddy’s hot plate the ability to develop diabetes induced thermal hyperalgesia was completely inhibited in rats treated orally with aminoguanidine as compare to diabetic rats. The radiant heat analgesiometer test showed increase in the latency of the tail flicking reaction in the aminoguanidine treated animals. In vascular reactivity study diabetes induced vascular dysfunction were found restored by administration of aminoguanidine in diabetic rats.

**Conclusion:**

Aminoguanidine restored diabetes induced hyperalgesia and vascular dysfunction.

**502**

**The declaration of Istanbul: A controversial issue in medical ethics**

Bansal A, Jain C, Bhargava M

**SMS medical college, Jaipur, Rajasthan, India.**

**Introduction:**

To respect the human dignity and protect the vulnerable, various laws and code of medicine, medical ethics were formulated. Latest addition to this list is declaration of Istanbul, 2008 for organ trafficking and transplant tourism. Live-donor transplantation saves thousands lives every year whoever may be the donor. But it has also increased commercialization of this noble act. This study was conducted to create the awareness and study the views of medical personnale related to organ transplantation in current scenario of globalization.

**Materials and Methods:**

Eighty faculty members and post graduates were included in this study. A questionnaire was developed to assess their basic knowledge regarding medical ethics and related landmarks. They were asked for their opinion about role of Pharmacologists in ethical society and transplantation team. Their views related to transplant tourism, organ trafficking, live donor organ transplantation its pros-.cons and transplant commercialization with various suggestions for preventing commercialization were also recorded.

**Result:**

The study show 72 participants were known to medical ethics, its importance and historical aspects But only 10 were familiar to DOI. 64 affirmed the need of pharmacologists in ethics and transplantation team and only 28 could differentiate between trafficking, tourism, and commercialization. When explained DOI, 64 supported its relevance and importance. While 16 believed that demands should be met irrespective of source. Details will be discussed.

**Conclusion:**

80% found DOI should be strongly advocated but 20% thought that forming stringent laws to totally ban inter-territorial organ transplant will only widen the gap in demand-supply.

**503**

**Non-prescription usage of antibiotics: A survey analysis**

Ved J

**Topiwala National Medical college and B.Y.L. Nair charitable hospital, Mumbai, India.**

**Introduction:**

The threat of resistance over the endangered Antibiotic era needs a unanimous defense from all the sections of society. It is essential to recognize simple things like justifiable use of Antibiotics to make this effort possible.

**Objective:**

To analyse the non-prescription usage of Antibiotics, its prevalence and trends, among patients.

**Methods:**

Survey was carried out among residents (25) of clinical departments of Nair hospital, Mumbai. Information was collected on the prevalence, causes and dosage patterns of the over-the-counter (OTC) purchase of Antibiotics by patients. Survey was extended to Chemists and an educated subgroup (100) of general population. Weighted mean values were calculated and analysed.

**Results:**

15-20% patients consumed nonprescribed antibiotics. Most common causative illness was Upper respiratory tract infections (81.17%). Amino-Penicillins (39.67%) and Azithromycin (35.34%) were the most common Antibiotics purchased without prescription. <15% of patients completed the full course of Antibiotic treatment. Most common reason for OTC purchase was previous successful experience of patients, followed by Chemist’s advice. Similar findings were present among the educated subgroup of population.

**Conclusion:**

>80% of OTC antibiotics are purchased for URTIs, 90% of which are selflimiting Viral illnesses. >85% of patients consume antibiotics till symptomatic relief occurs; to avoid unncecssary costs/side-effectsor due to ignorance. Incidence of OTC use of Azithromycin and Fluoroquinolones is increasing, indicating spreading resistance to Amino-Penicillins. Prevalence and usage patterns were similar in educated subgroup, very much suggestive of lack of awareness.

**504**

**Testing of drug samples: A trend analysis**

Jai Prakash, Singh GN

**Central Indian Pharmacopoeia Laboratory, Sector-23, Raj Nagar, Ghaziabad, India.**

**Objective:**

To report the trend of analysis of drugs samples for their quality during the last 5 years.

**Materials and Methods:**

The samples (both the raw material and finished products) received in the Sample Warden Section were distributed to the analysts in different divisions of the laboratory for their analysis. The main test performed were identification, assay (chemical microbiological), disintegration, dissolution, uniformity of weight, bacterial endotoxin test and or pyrogen test, sterility, abnormal toxicity etc. Other tests comprised the physical characteristics and labelling. The working reference standards served the purpose of comparison of results. The report of each sample was generated by compiling all the relevant results and a decision was taken regarding the quality of the sample and the concerned sample forwarding authority was informed.

**Results:**

The total number of 7693 samples were analysed during the last 5 years (2003-2004, 2004-05, 2005-06, 2006-07, 2007-08). The maximum number of samples were analysed in the year 2006-07 (including rapid assessment survey samples) and minimum number during the year 2005-06. The samples found of substandard quality in above periods were 6.2%, 7.5%, 7.1%, 1.7% and 2.9% respectively and that of spurious quality were 0.7%, 0.9%, 0%, 0.2% and 0.5% respectively.

**Conclusion:**

The trend of analysis of drug samples revealed only low percentage of substandard and spurious quality throughout the 5 years except that no spurious sample was reported during 2005-06. Strict vigilance and enforcement of law will help in improving the quality of the medicines in the country. The Indian Pharmacopoeia Commission is working to update the standards by regular publication of IP and providing Indian Pharmacopoeia Reference Substances (IPRS) to the stakeholders that would lead to further improvement in the quality of drugs and pharmaceuticals.

**505**

**Evaluation of *Coleus amboinicus* for anti-epileptic and anti-oxidant activity**

Pushpa Kumari B, Ranganayakulu D

**Sri Padmavathi Shool of Pharmacy, Tiruchanoor, Tirupathi, India.**

**Objective:**

Conventional antiepileptic drugs have been an effective tool in preventing and treating epileptic seizures. Inspite of theirefficacy, the drugs possess serious adverse effects which are more promising than the disorder and thereby impair the quality of life of patients. The recurrent episodic seizures and the prolonged use of antiepileptic drugs induce oxidative stress. So, it was considered interesting to do work on a herbal folklore medicine which may be potent against epilepsy and prevents the formation of free radicals. The study also aims to improve the treatment of epilepsy by means of vegetable source with no or less side effects.

**Materials and Methods:**

Epilepsy was induced by means of maximum electric shock (150mA for 0.2sec) in male albino rats with atleast 5 animals in each group. One group was kept as control and others received phenytoin as standard and fresh leaf juice of *Coleus amboinicus*. The drug was administered for 45 days continuously to study the impairment of normal behavior and memory of rats if any, because conventional antiepileptic drugs bring about cognitive and behavioral deficits. Various behavioral parameters such as locomotor activity, motor coordination test and cognitive test using Morris water maze were evaluated. At the end of the study, anti oxidant effect was studied by qualitative estimation of catalase, SOD and lipid peroxidation in brain tissue homogenate.

**Results:**

ANOVA was employed for statistical analysis. Fresh leaf juice of Coleus amboinicus has shown potent antiepileptic and anti oxidant activity.

**Conclusion:**

The results obtained in the study can be taken as a basis for further investigation and determination of the exact constituent responsible for these activities and their mechanisms of action.

**506**

**Dasmool kwatha: Effect on the analgesic activity of tramadol in rats**

Gehlot A, Mathur AK, Chouhan O

**Department of Pharmacology, Dr. S.N. Medical College, Jodhpur, India.**

**Objective:**

To study the effect of different doses of Dasmool Kwatha on the analgesic activity of Tramadol.

**Methods:**

Experiments were carried out on ‘albino rat model’ using tail flick method. The animals were treated with different doses of Dasmool Kwatha (0.5g/100g, 1.0g/100g and 5.0/100g, orally). Finally combination of non analgesic doses of Tramadol (10mg/kg) with Dasmool Kwatha under study were administered to find out whether these combinations provided any additive/synergistic effect on nociceptive activity.

**Results:**

Dasmool Kwatha in a dose of 1.0g/100g, found to highly significant analgesic. Tramadol showed no antinociception action when given in a dose 10 mg/kg i.p., but it showed excellent antinociception by increasing the tail flick latencies at affective dose (25 mg/kg i.p.).

**Conclusion:**

From the present study it may be concluded that Dasmool Kwatha and Tramadol have potential synergistic effect on nociceptive activity.

**507**

**Evaluation of stability and *in vivo* trans-corneal penetration of 1% topical calcium dobesilate formulation**

Nirmal J, Velpandian T, Ghose S

**All India Institute of Medical Sciences (AIIMS), New Delhi, India.**

**Introduction:**

Calcium dobesilate (CDO) is a potent antioxidant, vaso-protector, veno-tonic, inhibit polyol pathway and glycation of proteins. A new topical formulation of 1% CDO has been developed and evaluated for its stability at different temperatures and *in vivo* trans-corneal penetration in rabbits.

**Methods:**

The stability study of the above formulation was carried out. For conducting trans-corneal penetration study, New Zealand albino rabbits of either sex weighing 1.5-2.5 Kg body were procured from the central animal facility, AIIMS. Fifty micro liters of 1% sterile CDO formulation was instilled into cul-de-sac of rabbit using calibrated pipette. At different time intervals (15, 30, 60 and 120 minutes), 50-75 *µ*l of aqueous humor was aspirated under topical anesthesia (4% xylocaine). Four eyes were used for each time point. All collected samples were stored at -70°C till analysis by HPLC.

**Results:**

The mean concentration of CDO present after three month of storage was found to be 91.89, 93.88, 100.5, and 94.89% respectively at 40°C, Room temperature, 4°C and -20°C. The transcorneal penetration study resulted the maximum mean concentration of 0.749±0.08 *µ*g/ml at 30 minute following single topical instillation.

**Conclusions:**

From this study, we conclude that the formulation was found to be more stable at 4°C and CDO showed predictable trans-corneal penetration in rabbits.

**Acknowledgement:**

We acknowledge Ozone Research Foundation (Ozone pharmaceuticals) for providing financial grant for supporting this research work.

**508**

**Evaluation of herbomineral preparation (*Dolabi*) in diabetic neuropathy**

Shaikh AS^1^, Gawali VB^1^, Singhai AK^2^, Somani RS^1^

**^1^Sinhgad College of Pharmacy, Pune, India, ^2^Dept. of Pharm. Sci., Dr. HS Gour University, Sagar, India.**

**Objective:**

Neuropathic pain is an important complication of diabetes and clinical studies have reported difficulty in managing diabetic neuropathy. Many indigenous herbominaral preparations have been reported to lower blood sugar level in diabetic individuals. The present investigation aims to evaluate the Unani herbomineral preparation *Dolabi* for its role in diabetic neuropathy in rats.

**Method:**

Wistar rat were injected with STZ (55mg/kg, i.p.) to produced experimental diabetes. The herbomineral preparation (*Dolabi*) was administered in diabetic rats for six weeks. The changes in body weight were assessed after every two weeks treatment. Thermal hyperalgesia were studied for every two week for 4 week using water immersion test, cold allodynia at 4^th^ and 6^th^ week of diabetes using water immersion test and motor in co-ordination test was carried out after every week for 4 week of treatment using walking function test. The results of *Dolabi* treatment were compared with diabetic control and normoglycaemic animals. The blood glucose was estimated using glucometer.

**Result:**

Diabetic rats treated with *Dolabi* showed significant (*P*< 0.05) reduction in blood glucose level, delay in tail withdrawal in both thermal hyperalgesia and cold allodynia. Also motor co-ordination was improved as compared to diabetic control group.

**Conclusion:**

Thus, the *Unani* herbomineral preparation *Dolabi* has potential effect in diabetic neuropathy and warrants the need for further studies to elucidate its mode of action.

**509**

**Genetically modified foods: Are they safe: Biosafety regulations in India**

Saini Prem^1^, Anand Pinky^2^

**^1^B.S.Anangpuria Institute of Pharmacy, Faridabad, India, ^2^Supreme Court of India, New Delhi, India.**

Overall the revolution that is presently trying to overturn 12, 000 years of traditional and sustainable agriculture was launched in 1980 in the US. This was the result of a little-known US Supreme Court decision Diamond vs. Chakrabarty where the highest court decided that biological life could be legally patentable. Restructuring nature was not part of the bargain…this direction may be not only unwise, but dangerous. Potentially, it could breed new animal and plant diseases, new sources of cancer, novel epidemics. In 1989, dozens of Americans died and several thousands were afflicted and impaired by a genetically altered version of the food supplement – L-tryptophan. Widespread concerns have been expressed by scientists about the effects of GM foods on the environment and consumer health particularly allergnicity, antibiotic resistance, toxicity, ethical issues etc. As per WHO “different GM organisms (GMO) include different genes inserted in different ways. This means that individual GM foods and their safety should be assessed on a case-by-case basis and that it is not possible to make general statements on the safety of all GM foods”. The fundamental rights of safe, healthy, nutritious food and free from drugs, disease and contaminations are guaranteed under Article 19 and 21 of the Constitution of India including Protection of Food Adulteration Act, 1955; Consumer Protection Act, 1986; Environmental Protection Act, 1986. However, presently, there is no systematic, regulatory or legal framework to test, assess, evaluate and regulate GM Foods and their effects on human health.

**510**

**Comparision of efficacy of metformin vs N-Acetyl cysteine in patients of polycystic ovarian disease**

Singal R, Walia R, Sehgal V, Mohi MK, Bedi GK

**Government Medical College and Rajindra Hospital, Patiala, India.**

**Aims and Objective:**

To determine the efficacy of metformin vs. N-acetyl cysteine (NAC) on insulin resistance and BMI in patients with PCOS.

**Design:**

Prospective randomized open parallel group comparative study.

**Materials and Methods:**

Participants included 60 women diagnosed with PCOS randomized into two groups A and B of 30 women each. Group A of 30 women was put on metformin 1000-1500mg in divided doses for 6 months and group B of 30 women was put on N-acetyl cysteine 1.2-3 gm in divided doses for 6 months. They were assessed at 0, 3, 6 months. Decrease in insulin resistance was measured by HOMA-IR (Homeostasis Model Assessment- Insulin Resistance) method and also decrease in BMI was measured.

**Results:**

In Group A mean HOMA-IR at baseline was (3.77±0.66) and mean and percentage change at 6 months was (2.5±0.22, 30.4%) and BMI at baseline was (27.17 ±3.81), at 6 months mean and percentage change was (26.52±3.72, 2.4%). In group B HOMA-IR at baseline was (3.56±0.73) and mean and percentage change at 6 months was (3.55±0.68, 0.0316%) and BMI at baseline was (26.98±2.7), at 6 months mean and percentage change was (26.88±2.61, 0.33%).

**Conclusion:**

Change in BMI and HOMA-IR at 6 months was statistically significant with metformin treatment and there was no statistically significant change in BMI and HOMA-IR with NAC treatment in PCOS. Thus metformin is more efficacious in lowering BMI and insulin resistance than NAC.

**511**

**Hypoglycemic activity of dried seeds of *Acacia tortilis***

Agrawal NK, Kumar S, Singh SP, Singh S, Jain IP

**G.S.V.M. Medical college, Kanpur and N.B.R.I., Lucknow, India.**

**Objective:**

To analyze the hypoglycemic activity of freeze dried gum of seeds of Acacia tortilis.

**Materials and Methods:**

Dried seeds of *Acacia tortilis* were obtained from desert areas of western Rajasthan. These were grinded and sieved. Nonvolatile fraction of this extract is precipitated with ethyl alcohol and purified with the help of filtration technique followed by ion-exchange and freeze drying process. The study was carried out in albino rats weighing 150-200 gm divided into two groups each comprising of six rats. Group I served as Normal control received 2 ml distill water for 7 days and on 7^th^ day, Oral glucose tolerance test (OGTT) was done by administration of 2gm/kg glucose load, dissolved in distill water whereas group II served as Test group received 100mg/kg extract of freeze dried gum of *Acacia tortilis* for 7 days and on 7^th^ day, OGTT was done by administration of 2gm/kg glucose load plus 100mg/kg extract of *Acacia* dissolved in distill water. Blood glucose level was measured at 0 hr (after overnight fasting) followed by 1 hr and 2 hr (in OGTT).

**Results:**

At 0 hr and 2 hr blood glucose level significantly decreased (*P* < 0.05) whereas at 1 hr this effect was insignificant (*P* > 0.05) in group II.

**Conclusion:**

This study reveals that freeze dried gum of seeds of *Acacia tortilis* possess hypoglycemic activity and it should be further evaluated.

**512**

**Evaluation of *Acacia catechu* willd against selenite induced cataract in experimental animals**

Detroja J, Patel S, Patel K, Gandhi T

**Anand Pharmacy College, Anand, India.**

**Introduction:**

Sodium selenite is known to induce cataract through oxidative stress. *Acacia catechu* Willd. Possesses flavanoids and tannins, which have good antioxidant potential. Therefore the present study was undertaken to evaluate *A. catechu* Willd against selenite-induced cataract in experimental animals.

**Materials and Methods:**

Wistar suckling rats of either sex (40–50 gm) were randomly allocated to four groups (n=6). Except group I (normal control), cataract was induced in Group II(selenite control); Group III (standard) and Group IV(test) using a single dose (0.1ml) of 0.02 M sodium selenite administered S.C. to animals on 10^th^ day of life. Group III and IV additionally received Vitamin E (36 mg/kg; P.O; once daily) and ethyl acetate extract of *A. catechu* (500 mg/kg; P.O; once daily) starting from 15^th^ day of life for 18 days. Changes in biochemical parameters like total protein content, relative (insoluble and soluble) protein content, reduced glutathione content, sulfhydryl content, malondialdehyde level and calcium levels were observed in all group.

**Result:**

Sodium selenite induced significant decrease in total protein, relative protein, reduced glutathione, sulfhydryl content and increase in malondialdehyde and calcium level in the lens was significantly prevented by treatment with ethyl acetate extract of *A. catechu* and vitamin E.

**Conclusion:**

Anticataract activity of ethyl acetate extract of *A. catechu* can be attributed to decreased oxidative stress, which might be because of the presence of antioxidant constituents like flavanoids and tannins.

**513**

**Evaluation of wound healing activity of *Tridax procumbens* with honey**

Jarag RJ, Gubbi SR, More HN

**Bharati Vidyapeeth College of Pharmacy, Near Chitranagari, Kolhapur, (M.S), India. 416013**

The present study was aimed to evaluate the wound healing activity of extracts of leaf parts of *Tridax procumbens* in combination with Honey. It is a well-known plant in Indian traditional medicine. On the basis of traditional use and literature references, this plant was selected for evaluation of wound healing activity. An alcoholic and aqueous extracts of leaf part of *Tridax procumbens* was examined for wound healing activity in the form of ointment in three types of wound models on albino rats: the excision, the incision and dead space wound model. The extracts ointments showed considerable difference in response in all the above said wound models as comparable to those of a standard drug nitrofurazone ointment (0.2% w/w NFZ) in terms % closure, time of epithelization, scar size, tensile strength. Also the combination of *Tridax procumbens* and Honey showed significant increase in wound healing property for all three model compared to plane extract formulations. Stability study showed that formulation with honey is more stable than plane extract formulations. The alcoholic extract of plant with Honey showed maximum wound healing activity compared to other formulations. The histopathological examination of the granuloma tissue showed increased collagenation, which confirms the wound healing activity of *Tridax procumbens* with Honey.

**514**

**Antagonism of serotonergic system potentiates the effect of alpha-melanocyte-stimulating hormone on locomotor recovery after experimental spinal cord injury**

Rangani RJ, Bharne AP, Upadhya MA, Kokare DM, Dandekar MP, Subhedar NK

**Department of Pharmaceutical Sciences, RTM Nagpur University, Campus, Amravati Road, Nagpur, India.**

**Objective:**

While spinal cord injury (SCI) is a distressing condition, promising therapeutic interventions are largely missing. Melanocortin and serotonergic (5-HT) systems are known for their vitality in the regulation of neuronal regeneration and locomotor recovery following experimental SCI (ESCI). This prompted us to explore the involvement of serotonergic system in alpha-melanocyte stimulating hormone (alpha-MSH) induced locomotor recovery following ESCI.

**Materials and Methods:**

ESCI was induced by compression method at thoracic T_10-12_ level in Swiss-albino mice. While alpha-MSH was administered into fourth ventricle, ritanserin was given via intravenous route alone as well as in combination with the peptide. Each mouse was subjected to motor function score (0-10) before ESCI, and on day 1, 4, 7, 10 and 14 post-injury.

**Results:**

Administration of alpha-MSH (0.5-2 μg/mouse, for 2 weeks following injury) directly into the fourth ventricle, dose dependently improved the locomotor recovery. Treatment of 5-HT_2a/2c_ receptors antagonist ritanserin (0.1-1 mg/kg, intravenous) immediately following ESCI prevented the locomotor dysfunction observed on day 1, and also on 4, 7, 10 and 14 post-injury time points. Moreover, ritanserin (0.5 mg/ kg) potentiated the effect of alpha-MSH (0.5 and 1.25 μg/mouse).

**Conclusion:**

These results suggest that 1) immediate antagonism of 5-HT_2a/2c_ receptors following ESCI may reduce the early neurotoxic effects of 5-HT at injury site, 2) alpha-MSH alone may show neuroprotection and axonal regeneration property, and 3) the two agents may act synergistically and produce improvement in locomotor recovery.

**515**

**Influence of dapsone on cutaneous wound healing in male Wistar rats**

Chandrashekar K, Hogade AP, Hiremath SV, Patil PA

**J.N.Medical College, Belgaum, India.**

**Objectives:**

Dapsone has been reported to promote healing in brown resclue spider bite and also in doxorubicin caused extravastion injuries. Despite its reported anti-inflammatory property and antileprotic property paucity of information with respect to wound healing with this regard, the present study was planned to investigate the influence of dapsone on incision, excision and dead space wound in male wistar rats.

**Methods:**

Excision wounds as described by Morton and Malone, resutured incision wounds by Ehrlich and Hunt and dead space wounds by modified D Arcy technique were inflicted under light ether anesthesia with aseptic precaution to male wistar rats weighing 150-250g. Minimum six animals were used in each of the models and drugs were given orally once a day for 10 days in resutured incision and dead space wound groups, while treatment continued till complete epithelization in excision wound groups.

1. Closure of excision wounds was monitored by planimetry. Scar shape and size were noted on complete epithelization.
2. Healing of 10 days old resutured incision wounds was assessed by wound breaking strength.
3. Healing of 10 day old dead space wounds was assessed by granuloma dry weight, granuloma breaking strength, granuloma hydroxyproline content and granuloma histopathology.

**Results:**

As compared to controls dapsone significantly enhanced healing in all the wound models. Hydroxyproline contents and histological studies of the granulation tissue confirmed the influence of the dapsone on wound healing.

**Conclusion:**

Pro healing property of dapsone needs to confirmed clinically.

**516**

**A low molecular weight antineoplastic protein (BMP1) from the common Indian toad skin extract**

Bhattacharjee P, Gomes A

**University of Calcutta. 92, A P C Road, Kolkata, India.**

**Introduction:**

Amphibian skin is a treasure house of various bioactive molecules having therapeutic potentials. In the present study, a protein antineoplastic factor(s) was identified from the aqueous skin extract of the common Indian toad *Bufo melanostictus* Schneider.

**Methods:**

Toad (*Bufo melanostictus* Schneider) skin aqueous extract (TSAE) was prepared in distilled water and was expressed in terms of protein. A protein (BMP1) was purified by DEAE -IEC and reverse phase HPLC. Molecular wt. was determined by SDS-PAGE. Anticancer activity was done on U937 and K562 cell lines and EAC mice.

**Results:**

TSAE applied on DEAE-cellulose column, produced four protein peaks. Protein peak eluted with 0.02M NaCl designated as BMP1. BMP1 applied on RP-HPLC produced a major peak with retention time of 2.8 min. Molecular wt. of BMP1 was found to be 8KD. The yield of BMP1 was about 3 ± 0.72% of toad skin extract. IC 50 Value of BMP1 on K562 is 2.9 *µ*g and on U937 is 5.4 μg. BMP-1 produced significant inhibition in MTT value in K562 and U937cells. BMP1 produced significant inhibition of EAC cell count as compared to control. Further structural and functional study with BMP1 is in progress.

**Conclusion:**

It may be concluded that, A low molecular weight the protein (BMP1) was purified form toad skin extract that possess cancer cell killing property. Further detail studies on this protein in progress.

**517**

**Evaluation of the efficacy of Hajrul Yahud Bhasma in urolithiasis using rat as an experimental model**

Rao N, Shah J, Patel K, Gandhi T

**Anand Pharmacy College, Anand, India.**

**Objective:**

To evaluate antiurolithiatic activity of Hajrul Yahud Bhasma in urolithiasis using rat as an experimental model.

**Materials and Methods:**

Male Wistar albino rats (250-300g) were selected and divided into five groups. Except group I (normal) and group-II (Sham operated), in group-III (model control), group-IV (standard) and group-V (test) urolithiasis was induced by Calcium oxalate seed deposition in bladder surgically (3 mm diameter). Cystone (750 mg/kg, p.o.) and Hajrul Yahud Bhasma (100 mg/kg and 200 mg/kg, p.o.) were administered to group-IV and group-V for 14 days after surgery respectively. X-rays were taken before and after the treatment. At the end of treatment, blood and 24 urine samples were collected. Stones were collected and % matrix growth was evaluated. Several physical parameters (body weight, water intake, diuresis), stone forming promoters (calcium, oxalate, inorganic phosphate and uric acid) and inhibitors (magnesium, citrate) were analyzed. Scanning Electron Microscopy (SEM) and stereomicroscopy were performed on the stones.

**Results:**

Increase in % matrix growth, stone forming promoters and decrease in physical parameters and stone forming inhibitors induced by surgical implantation of Calcium oxalate seed was significantly reversed by treatment with Hajrul Yahud Bhasma. X-ray, SEM and steromicroscopy showed significant decrease in growth of crystals of Calcium oxalate implanted surgically on treatment with Hajrul Yahud Bhasma.

**Conclusion:**

This antiurolithiatic activity of Hajrul Yahud Bhasma may be because of its ability to increase inhibitors’ level and decrease promoters’ levels.

**518**

**Evaluation of anti-ischemic activity of quinapril and lycopene in hepatic ischemia reperfusion injury in rats**

Patel VM^1^, Tripathi P^2^, Patel NJ^2^, Patel DG^3^

**^1^Shree Krishna Institute of Pharmacy, Mehsana, India, ^2^S.K.Patel College of Pharmaceutical Education and Research, Mehsana, India, ^3^Radhe School of Pharmacy and Bio-Research Institute, Mehsana, India.**

The study was designed to study the effects of Quinapril (QP) and Lycopene (LYC) on hepatic ischemia reperfusion (HIR) injury in rats. For this purpose Wistar albino rats were subjected to 45 minutes of hepatic ischemia followed by 3 hrs reperfusion period. Animals were divided in to normal control, sham control, HIR, QP (1.5, 3, 5 mg/kg) + HIR, LYC (10 mg/kg) + HIR and a combination (QP + LYC) + HIR groups. Serum aspartate aminotransferse (AST) and alanine aminotransferase (ALT) levels were determined to assess liver functions. Liver tissues were taken for determination of malondialdehyde (MDA), superoxide dismutase (SOD), catalase and glutathione (GSH) levels. Plasma AST and ALT activities were higher in HIR group than in control. They were decreased in the groups given QP, LYC or the combination. Hepatic GSH, SOD and CAT levels significantly depressed by HIR, were elevated to control levels in the combination group, where as treatment with QP or LYC alone provided only a limited protection. Hepatic MDA levels were significantly increased by HIR. This increase in MDA was partially decreased by QP or LYC alone, whereas treatment with the combination reduced these values back to control levels. In conclusion, considering the dosage used, LYC appears to be significantly more potent than QP in reversing the oxidative damage induced by HIR. Our findings show that LYC and QP have beneficial effect against the HIR injury and due to their synergistic effects, when administered in combination, may have more pronounced protective effect on the liver.

**519**

**Evaluation of the efficacy of *Citrus medica* linn against ethylene glycol induced urolithiasis in rats**

Chauhan K, Patel S, Patel K, Gandhi T

**Anand Pharmacy College, Anand, India.**

**Objective:**

To evaluate the efficacy of *Citrus medica* Linn against ethylene glycol induced urolithiasis in rats.

**Materials and Methods:**

Male Wistar albino rats weighing 250-300g were randomly allocated to four groups (n=6). Except Group I (normal), Group II (model), III (std), IV (test), received Ethylene glycol (0.75% EG in drinking water; 28 days) for induction of urolithiasis. Group III and IV additionally received Cystone (750 mg/kg, p.o.), *Citrus medica* (250 mg/kg, p.o.) respectively for 28 days. After the completion of treatment, blood and 24 hr urine samples were collected. Body weight, diuresis and urine pH were measured. Isolated kidneys were weighed (wet and dry) and were used for histopathology study as well as homogenate preparation. Various stone forming inhibitors (citrate and magnesium) and promoters (calcium, oxalate, inorganic phosphate, uric acid) were analyzed in urine, serum and kidney homogenate. Renal function test, antioxidant parameters and crystalluria were also evaluated.

**Result:**

Induction of urolithiasis by ethylene glycol *caused* significant increase in kidney weight (dry and wet), crystalluria and various stone forming promoters, and decreased urine volume, body weight, and various stone forming inhibitors. Stone formation significantly deteriorated renal function and caused oxidative stress. These changes were significantly prevented by treatment with *Citrus medica* and Cystone. Urinary pH was also altered from alkaline to neutral. Histopathological results were also consistent with above findings.

**Conclusion:**

***Citrus medica*** showed significant activity against urolithiasis which may be because of its diuretic activity, ability to increase inhibitors level and decrease promoters levels.

**520**

**Inorganic nanoparticles based gene delivery system**

Naqvi S, Akmal M, Abdin MZ, Samim M

**Jamia Hamdard, Hamdard Nagar, New Delhi, India.**

**Introduction:**

Gene therapy promises a new evolution in medicine by correcting desired phenotypes and produced engineered DNA that carries a therapeutic gene that can be introduced into somatic cells and ultimately produced therapeutic effects. Viral vectors have several practical and conceptual problems such as host immunogenic response, high cost, lack of sustained expression, cytotoxicity limited DNA carrying capacity and production. To overcome these problems we prepared inorganic plasmid loaded calcium phosphate nanoparticles for better transfection efficiency.

**Methods:**

[1] plasmid isolation using alkaline lysis method.[2] Preparation of loaded and void calcium phosphate DNA using reverse micelle method. [3] The physiochemical characteristics were studied using X-ray diffractrometery, TEM, dynamic light scattering (DLS). [4] Maximum entrapment efficiency was determined by gel electrophoresis and U.V spectrophotometer. [5]Release profile of pDNA and endosomal escape studies was done with the help of the gel electrophoresis. [6] In vitro transfection studies were done using *Brassica juncea* hypocotyl explants by GUS assay.

**Results:**

TEM results of void and encapsulated calcium phosphate nanoparticles revealed the formation of dense particles of size smaller than 50nm with spherical morphology as well as narrow size distribution. X-ray diffractrometery showed its crystallinity having hydroxyappetite structure. Entrapment efficiency determination studies using U.V spectrophotometery and gel electrophoresis showed 99% entrapment of DNA in calcium phosphate nanoparticles. *In vitro* transfection studies showed high transfection as compare to conventional methods such as agrobacterium mediated gene delivery, gene gun method etc.

**Conclusion:**

Calcium phosphate nanoparticles have high transfection efficiency as compare to conventional gene delivery methods and it proves to be more effective non–viral vector in gene therapy. DNA entrapped into these nanoparticles is protected from the extra cellular enzymatic degradation. These particles cause osmotic disbalance with consequent disruption of the endosome and this enables the DNA to come out into the cytosol.

**521**

**Evaluation of the effect of ethyl acetate extract of cutch (*Acacia catechu*) on glucose and hydrocortisone induced hyperglycemia in albino rats**

Gunindro N, Krishnapramodini K, Imoba ST

**Regional Institute of Medical Sciences, Imphal (Manipur), India.**

**Introduction:**

Despite the reports on hypoglycemic property of *Acacia catechu* (family-leguminosae), the underlying mechanism remains undocumented. We studied effect of cutch, a product of *Acacia catechu* on hydrocortisone and glucose induced hyperglycemia to assess its effects on gluconeogenesis and insulin secretion.

**Methods:**

Effect of ethyl acetate extract of cutch (EAC) on glucocorticoid induced hyperglycemia (hydrocortisone sodium succinate-250mg/kg i.p) was tested in different groups (control, standard, test-I and II) of healthy Wistar albino rats of either sex (n=6). To study effect on glucose induced hyperglycemia (D-glucose- 3g/kg p.o), a single group was used as control, standard, test-1 and II groups successively with drug wash out period of 10 days. Control groups received 2% gum acacia in D/W (1ml/100g p.o). Standard groups in the glucocorticoid and glucose induced hyperglycemia received metformin (13.5 mg/100g) and nateglinide (1.6mg/100g) respectively. Test-I and II groups were administered 250 and 500mg/100g of EAC. Standard and test drugs were prepared in 2% gum acacia suspension, and administered (1ml/100g) per orally. Changes in mean serum glucose at 1 and 2h of treatment from pretreatment fasting values in the different groups were compared. Results were analysed by One- way ANOVA followed by Bonferroni test.

**Results:**

Metformin and nateglinide significantly reduced glucose in both hydrocortisone and glucose induced hyperglycemia. Only hydrocortisone induced hyperglycemia was reduced significantly (*P*<0.05) by EAC.

**Conclusion:**

Antihyperglyemic effects of metformin and nateglinide could be seen. Reduction of serum glucose by EAC is attributed to its inhibitory effect on gluconeogenesis. EAC is unlikely to be an insulin secretagogue.

**522**

**Evaluation of the anti-cataract potential of a polyherbal formulation (Itone)**

Gupta P, Velpandian T, Sharma C, Ravi AK, Biswas NR, Ghose S

**All India Institute of Medical Sciences (AIIMS), New Delhi, India.**

**Purpose:**

To evaluate the anticataract potential of a sterile polyherbal formulation (Itone) in various experimental models of cataract.

**Methods:**

Anti-cataract activity of Itone was evaluated in steroid induced cataract in developing chick embryos and selenite induced cataract in pups, after subcutaneous administration. Briefly, for the steroid induced cataract, ten days old fresh fertilized broiler eggs were purchased from a local hatchery and further incubated at 37°±2°C in a humidified incubator. Eggs were randomly divided into 3 groups having 4 each. Hydrocortisone was injected on the 15^th^ day followed by instillation of itone eye drops after 3, 10 and 17 hrs. The eyes were enucleated after sacrificing and subjected for cataract grading after 48 hrs. of hydrocortisone administration. For the selenite model, Wistar female albino rat pups were used. Sodium selenite was injected subcutaneously on the 9^th^ post-natal day. Itone was administered intraperitoneally from the day of selenite administration till 16^th^ day. The eyes were further subjected for examination under the slit lamp for the grading of cataract.

**Results:**

In the hydrocortisone induced cataract, Itone treated lenses were graded for stages 3 and 4, respectively. While in the selenite induced cataract model, Itone treated lenses were in stages 0, 1 and 3, in comparison to the control lenses (stage 4).

**Conclusions:**

From this study, we conclude that the sterile polyherbal formulation may have some beneficial role in delaying the progression of cataract.

**523**

**Evaluation of activity of *Acacia catechu* willd. against galactose induced cataract in rats**

Gajera V, Patel S, Patel K, Gandhi T

**Anand Pharmacy College, Anand, India.**

**Objective:**

To evaluate activity of *Acacia catechu* Willd. against galactose induced cataract in rats.

**Materials and Methods:**

Male SD suckling rats were randomly allocated to four groups (n=6). Group I(normal) animals were fed with normal laboratory chow where as animals of Group II(model), III(standard), IV(test) were fed with galactose diet for 18 days starting from day 21 after parturition. Three days prior to the galactose feeding, treatment with quercetin(400 mg/kg) and ethyl acetate extract of *A.catechu* (500 mg/kg) was administered to group III and IV respectively for 21 days. Gross examination and microscopic evaluation was done every third day of treatment. All animals were sacrificed on 18^th^ day of galactose feeding, to estimate various biochemical parameters.

**Result:**

Gross examination and Microscopic evaluation of lenses indicated that treatment with ethyl acetate extract of *Acacia catechu* delayed the maturation process of galactose induced cataract. *Acacia catechu* pretreatment significantly prevented rise in aldose reductase activity, relative insoluble protein content, malondialdehyde and calcium level in the lens and significantly raised relative soluble protein, reduced glutathione and sulfhydryl content in lens induced by galactose rich diet.

**Conclusion:**

The anticataract activity of *A.catechu* may be attributed to decreased oxidative stress, reduced aldose reductase activity and prevention of alteration in lens membrane proteins.

**524**

**Clinical study of polyherbal formulation in allergic conjunctivitis**

Ravi AK, Chandra M, Ghose S, Velpandian T

**Department of Ocular Pharmacology and Pharmacy, Dr. R P Centre for Ophthalmic Sciences, All India Institute of Medical Sciences (AIIMS), Ansari Nagar, New Delhi - 110 029, India.**

**Purpose:**

To evaluate the efficacy of polyherbal formulation (ITIS) in patients suffering from allergic conjunctivitis.

**Methods:**

The study protocol was approved by the Institutional Human Ethics Committee, AIIMS. Twenty-one patients having allergic conjunctivitis of either sex were enrolled. Patients having any other ocular disorders or already undergoing treatment for allergic conjunctivitis and noncompliant were excluded from the study. Selected patients had undergone slit-lamp examination and severity was graded according to symptoms (redness, itching, watering). Patients were advised to instill one drop of ITIS three times a day in the affected eye. Nonresponders within one week of ITIS therapy were shifted to receive 0.1% olapatidine TDS.

**Results:**

This interim analysis included 11 males and 10 females having the mean age 30.24±16.82 yrs. 76% of patients responded well with ITIS eye drop and symptoms were resolved within a week. 23% of patients did not responded to ITIS, then they were shifted to receive olopatadine 0.1% and symptoms were disappeared in the following week of the treatment. Surprisingly, olapatidine non-responders (n=4) not enrolled in the present study recovered when shifted to ITIS in the following week.

**Conclusions:**

In the treatment of allergic conjunctivitis, polyherbal formulation (ITIS) is equally effective as well as olopatadine 0.1% eye drop. However, further studies are in progress to evaluate the effectiveness of ITIS in more patients.

**525**

**A fluorescence polarization- based, rapid, costeffective, generic and high throughput kinase assay**

Chopra P, Nanda K, Chatterji M, Bajpai M, Dastidar SG, Ray A, Bhatnagar P

**Ranbaxy Research Laboratories, Gurgaon, India.**

The pivotal role of kinases in signal transduction and cellular regulation has made them an attractive pharmacological target across a broad spectrum of human diseases. Small-molecule inhibitors of protein kinases are considered as novel class of drugs for therapeutic intervention. The focus of most of the pharmaceutical companies is to screen large libraries of compounds for their inhibitory potential against kinase of interest in high-throughput screening (HTS) format. In recent years, technique to identify kinase inhibitors by HTS has evolved rapidly. The *in vitro* kinase assays seem to be as ubiquitous as kinases themselves; therefore, selection of a kinase assay is a daunting task. HTS assay should be homogenous, cost effective, non-radioactive, generic and less time consuming. Therefore, there is a need to develop a generic cost-effective and rapid kinase assay, amenable to HTS. The present study provides a method of assaying protein kinase activity using zinc cocktail in a fluorescence polarization- (FP) based format. Assay conditions were standardized manually and then further validated in a HTS format using liquid handler. Results obtained in HTS assay system were compared with the commercially available fluorescence-based assay and both results showed very good agreement. In summary, present study provides an improved method of screening of protein kinase inhibitors in a homogenous, cost-effective, non-radioactive, rapid and high-throughput format.

**526**

**Evaluation of the activity of *Commiphora mukul* in the treatment of hyperlipidemia and atherosclerosis using experimental animals**

Shah T, Patel A, Patel K, Gandhi T

**Anand Pharmacy College, Anand, India.**

**Objective:**

To evaluate the activity of *Commiphora mukul* in the treatment of hyperlipidemia and atherosclerosis using experimental animals.

**Materials and Methods:**

Sprague Dawley rats (200-300 gm) were randomly allocated to 5 groups (n=6). Group I(normal) and II(vehicle control) received a standard rat chow diet; groups III (model control), IV (test), and V (standard) were fed with 20 gm/ rat/day high fat diet (cholesterol 2%, sodium cholate 1%, coconut oil 2.5%) for 28 days. Additionally group II, IV and V received Twin 80(5 ml/kg, p.o), ethyl acetate extract of Commiphora mukul (300 mg/kg., P.O) and Atorvastatin (15 mg/kg, p.o) respectively for 28 days. On 29^th^ day, blood collected by retro orbital puncture technique was used for the evaluation of lipid profile. For atherosclerotic model S.D. animals were divided into four groups and subjected to same treatment as above except standard group V. On the 8^th^ day of study lesion was produced through endothelial injury in the femoral artery in group III and IV. Femoral artery injury lesion index was measured on 29^th^ day.

**Result:**

High fat diet (HFD) caused significant increase in the serum cholesterol, triglyceride, VLDL-C, LDL-C and significant reduction in HDL-C level, which was significantly prevented by treatment with Commiphora mukul as well as by Atorvastatin. A decrease in femoral artery lesion index was observed with Commiphora mukul treated group as compared to model control.

**Conclusion:**

From the above results we concluded that Commiphora mukul is effective against hyperlipidemia and atherosclerosis.

**527**

**Student opinion on pharmacology teaching pattern at a tertiary care hospital**

Gangopadhyay T, Keshri USPD, Kumar Subodh, Sharma J

**Rajendra Institute Of Medical Sciences, Ranchi, Jharkhand, India.**

**Objectives:**

To evaluate student opinion about Pharmacology teaching pattern at a tertiary care teaching hospital.

**Methods:**

Students currently studying pharmacology were recruited randomly and they were given a pre-formed structured questionnaire and their responses were analyzed.

**Results:**

Themes that emerged on how the classes can be made more productive were to increase teacherstudent interaction, to give more stress on group discussions and tutorials, in-depth discussion on drug structure, biochemistry and physiology and to synchronize the lectures, tutorials and practicals.. The groups should be made smaller for better teacher-student interaction. Practical classes on prescription writing can be held in medical wards with real patients. The exams should be conducted more frequently with lesser number of topics. There is a general demand for publishing a year calendar at the beginning of the session.

**Conclusions:**

There is a need to make the classes more attractive and interactive and more clinically oriented.

**528**

**Pre-clinical investigation of nano-calcium-disodium-EDTA dry powder inhaler for ventilation lung scintigraphy**

Kumar N^1^, Mittal G^1^, Singh T^1^, Sultana S^2^, Ahmed F A^2^, Bhatnagar A^1^

**^1^Institute of Nuclear Medicine and Allied Sciences, Delhi, India, ^2^Jamia Hamdard University, New Delhi, India.**

The study was undertaken to determine clinical safety and efficacy of nano calcium-disodium ethylene diamine tetra acetic acid (Ca-EDTA) for ventilation lung scintigraphy. Ventilation lung scintigraphy is routinely used as diagnostic test for lungs. Presently it is done by nebulization with ^99m^Tc-DTPA or with pertechne gas. However, due to a high temperature requirement of about 2500°C and high cost, use of pertechne gas has its limitations. On the other hand, particle size of ^99m^Tc-DTPA used for the purpose ranges between 5-7 microns, with a substantial loss of drug that is exhaled back from the respiratory tree. Nano Ca- EDTA were prepared by controlled precipitation method and were characterized by Transmission electron microscopy (TEM), particle size analyzer (120-150nm) and scanning electron microscopy (SEM) studies were also carried out for the characterization of nano-formulation. A method for radiolabeling the optimized formulation of nano- Ca--EDTA with ^99m^Tc was standardized using reducing agent. The optimized radiolabeled formulation (labeling efficiency >95%) was then evaluated for its clinical safety and efficacy on healthy human volunteers for ventilation lung scintigraphy after the toxicological studies on albino rats and bio distribution in bulb ‘C’ mice. In vivo internalization of the nano-preparation was calculated in real time by taking radiolabeled Ca-EDTA in the nebulization chamber as 100% and activity in lungs and body as the internalized fraction in terms of percentage (>65%). Scintigraphy studies showed significant uptake of drug in the lungs. The results corroborated with the results obtained with tissue distribution studies done in animals. Ventilation lung scintigraphy with nano ^99m^Tc-Ca-EDTA in humans suggests acceptable imaging at 10 min.

**529**

**Prevalence of self medication among the urban educated people in Meerut**

Vishwakarma P^1^, Vishwakarma N^2^

**^1^L.L.R.M.Medical College, Meerut, India, ^2^Rishikul State Ayurvedic P.G.College, Hardwar, India.**

**Introduction:**

Practice of self-medication is more common in India due to slack implementation of drug-control act. We have conducted this study to assess the prevalence of self medication pattern among the urban educated people in Meerut city.

**Materials and Methods:**

This cross-sectional study was conducted in 250 urban families of bank-employees and teachers in Meerut city. Doctors and paramedics were excluded. A survey was conducted by using a preformed structured multirespons questionnaire and questions were asked about the self-medication practice, ailment for which it was used, source of information and knowledge about the dose, side-effects and drug-interactions.

**Results:**

About 60% of the study population was found to be practicing self medication without having any idea about the dose, duration and side effects. The most common ailment for self medication was pain with fever(84%) followed by cough and cold (62%) abdominal problems (55%) and allergy(48%). NSAIDs(89%) were used most commonly followed by cough and cold remedies(70%), laxatives and antacids(55%) and antihistaminics(35%).Sources of information were doctors (85%) friends or relatives (50%) and pharmacists (42%).Date of expiry, exact spelling and intactness of packaging was noticed by 60%, 35%, 10% of study population respectively. Reasons were found as minor ailments, OTC drugs, and high cost of doctor.s consultation fee.

**Conclusion:**

This study concludes in high prevalence of self medication among the bank employees and teachers in Meerut city, with lack of knowledge about the used drugs. Hence strict implementation of drug control act is must to prevent dangers of self medication in the society.

**530**

**First model list of essential medicines for children for rational prescribing**

Kastury N, Sharma A, Chugh Y

**MLN Medical College, Allahabad, India.**

Essential medicines are those that satisfy the priority health needs of the population. WHO published its first model list of essential medicines (EML) in 1977, which is being updated successively at an interval of 2-3 years, in view of the changing world scenario about the medical needs and availability of new and better drugs. In October 2007 first EML intended for use in children up to 12 years of age was drawn by WHO. Core list presents a list of minimum medicines that are most efficacious, safe and cost effective for priority conditions. Priority conditions are selected on the basis of current and estimated future public health relevance and potential for safe and cost effective treatment. The format of the 15^th^ Model EML has been retained but some sections like medicines to treat gout, antiparkinsonian, antianginal, antiglaucoma, contraceptives, oxytocics etc. have been deleted because they contain the medicines that are not relevant for children. The core list includes 285 medicines including 63 complementary agents. 15 fixed dose combinations have been approved for use in children whereas 21 medicines have been indicated that there is an age restriction on use of these medicines.

**531**

**Use of placebo: Knowledge, attitude and practice in medical practitioners**

Shah KN^1^, Vyas BM^1^, Panchal DJ^2^, Patel VJ^1^

**^1^Smt NHL Municipal Medical College, Ahmedabad, India, ^2^Accutest, Ahmedabad, India.**

**Introduction:**

Use of placebo is justified in some controlled clinical trials. However, use of placebo has also extended to routine clinical practice, the extent of use being unknown. The present study was undertaken to evaluate the practice and pattern of use of placebo among medical practioners and their knowledge and attitude regarding placebo.

**Methods:**

This is a questionnaire based study. A questionnaire related to use of placebo in clinical practice was prepared and validated. Three groups of prescribers, namely specialists, resident doctors and general practioners were personally approached with the request to fill up questionnaire (30 in each group). Data was analyzed with the help of SPSS version 15.

**Results:**

All the 90 practitioners returned the questionnaires (M= 83, F= 7; age: 24 to 59 years). 80 out of 90 respondents reported use of placebo. There was no statistically significant difference in frequency of prescribing placebo among three groups (*P*=0.153). While prescribing placebo resident doctors labeled it as ‘medicine‘ more frequently compared to general practitioners (*P*<0.05). Only 1 general practitioner believed that while prescribing patients should be informed that it is a placebo. 90% of prescribers believed that placebo is usually/sometimes effective. According to 57.8% of the doctors, it is appropriate to use placebo by experienced hands or when there is evidence of efficacy. Only 3.3% doctors believed that placebo should never be used.

**Conclusion:**

Most practitioners use placebos in clinical practice. Use of placebo is an integral part of therapeutic practice.

**532**

**Documentation of the ocular toxicity of Holi colors in the northern states of India**

Gayathri C, Velpandian T, Ghose S

**All India Institute of Medical Sciences (AIIMS), New Delhi, India.**

**Introduction:**

To document the ocular toxicity of Holi colors in the Northern states of India (New Delhi and Uttar Pradesh).

**Methods:**

A reporting form along with sterile swabs for color identification were sent to 36 hospitals in Uttar Pradesh and patients reported to the eye casualty of AIIMS were subjected for this analysis. After the Holi festival in March 2007, 26 sample swabs were received from UP, Delhi and 13 eyewash samples from Delhi. The eyewash fluids collected were subjected for solid phase extraction using C-18 cartridges. The column was conditioned with 2ml acetonitrile and 4ml of water. Under negative pressure the eyewash fluid was passed at the speed of 1ml/min. The trapped dye was subjected to water wash (4ml) followed by elution with 2ml of acetonitrile for identification and quantification by HPLC analysis. Total 164 color powders collected from various states of northern India was subjected for analysis using spectrophotometer and thin layer chromatography. The peak absorbance was matched with the absorption of known dye.

**Results:**

Various color powders showed the presence of synthetic dyes like malachite green, auramine O, methyl violet, rhodamine and orange II. The eye wash fluid showed the presence of malachite green and rhodamine. Thirteen swab/eyewash samples were positive for both malachite green and rhodamine. Eighteen samples positive for malachite green. Among all, malachite green caused severe form of ocular manifestations including conjunctival edema and epithelial defect.

**Conclusion:**

The present analysis showed that Malachite green and Rhodamine are the major causative agents for ocular toxicity in the festival of colors (Holi) in Northern India. We acknowledge the fellowship provided to Ms. Gayathri by DST under Young Women Scientist Societal Programme.

**533**

**Studies on comparative evalution of the effect of penetration enhancers on passive diffusion and iontophoretic transport**

Mahender K^1^, Srivastava AK^2^

**^1^Meerabai Polytechnic, Maharani Bagh, New Delhi, ^2^Banaras Hindu University, Varnasi, India.**

**Introduction:**

To perform a comparative evaluation for the effect of penetration enhancers on passive diffusion and iontophoretic transport.

**Methods:**

Timolol maleate was used as model drug in concentration 10mg/ml. There were six groups of formulations, first three groups had different concentrations of each dimethyl sulfoxide (10%, 20% and 30%); sodium lauryl sulphate (0.10%, 0.20% and 0.30%) and sodium deoxycholate (0.05%, 0.10% and 0.20%) and other three groups are the combination of different concentrations of the same three enhancers. The control group consisted of the same groups. To study the effect of penetration enhancers Albino rats skin was used. The pretreated skin was rinsed with buffer solution pH 8.0 and the *invitro* passive and iontophoresis transport studies were performed at 0.7mA/cm^2^ using platinum electrodes. The samples were analyzed by measuring the absorbance at 294nm on a Jasco UV-Vis Spectrophotometer. The changes of rat abdominal skin due to application of the permeation enhancers either alone or in combination with iontophoresis and passive diffusion by an optiphoto light microscopy was also performed.

**Result:**

The flux, permeability coefficient and enhancement factor were significantly higher in iontophoretic transport as compared to passive diffusion. This was further proved by histological findings which shows that changes in histology of rat skin causes the enhancement of drug penetration as compared to control group.

**Conclusion:**

Iontophoresis in conjugation with permeation enhancers had significant synergistic effect in terms of transport of Timolol maleate across skin, than passive diffusion.

**534**

**Evaluation of gastro protective effect of methanolic extract of *Jatropha gossyfolia*. Linn in rats**

Vijayakumar AR^1^, Ilavarasan R^2^, Venkataraman S^3^

**^1^C.L.Baid Metha College of Pharmacy, Chennai, India, ^2^Captain Srinvasamurthy Drug Research Institute for Ayurveda, Chennai, India, ^3^Dr.CLBMFPE and R, Thoraipakkam, Chennai, India.**

**Introduction:**

The present study evaluated the gastro protective effect of Methanolic Extract of Jatropha Gossyfolia (MEJG) on aspirin plus pylorus ligation induced ulcer model in rats.

**Methods:**

Wister rats either sex weighing 180 to 250g were divided into five groups of 6 animals each. Aspirin (200mg/kg, o.p.) administered in non fasted rats once daily for 5 days. Ranitidine and MEJG (100mg/ kg and 200 mg/kg) were administered orally 30min before each aspirin treatment whereas the control group received only 1% carboxy methyl cellulose (10ml/kg). Pylorus ligation was performed on the rats which were fasted 36 h on the sixth day after aspirin administration. Four hour later rats were sacrificed with excess of sodium pento barbitone. The stomach was dissected out after tying the oesophagal end; the stomach was cut open along the greater curvature and the contents were estimated for pH, volume, ulcer score, free and total acidity.

**Results:**

The pH (3.35±0.04), gastric juice volume (2.52±0.04), ulcer score (6.50±0.43), free acidity (41.48±0.32) and total acidity (63.5±0.56) were significantly decreased (*P*<0.001) in the MEJG treated groups as compared to the control treated animals.

**Conclusion:**

It is concluded that methanolic extract of *Jatropha gossyfolia* exhibited significant protection in aspirin induced ulcer model.

**535**

**Anti-diabetic activity of aqueous extract of *Vicia faba* seeds in streptozotocin-induced diabetic rats**

Joshi P^1^, Santani D^2^, Goyal R^3^

**^1^L. M. College of Pharmacy, Ahmedabad, India, ^2^Rofel College of Pharmacy and Research, Vapi, India, ^3^M. S. University, Baroda, India.**

Dopaminergic system regulates glucose and lipid metabolism. Since *Vicia faba* Linn. (*Fabaceae*) is reported to be rich in levodopa, we investigated role of aqueous extract of dried *Vicia faba* seeds (Aqueous Extract) in diabetes and associated renal complications in streptozotocin-induced type 1 diabetic wistar rats (STZ-diabetic rats) at 300 mg/kg, p.o. dose (30 days). As STZ-diabetic rats did not show significant change in the renal functions after 30 days, we extended our study to 60 days. Though there was no effect of Aqueous Extract on loss of body weight, polydipsia, polyphagia, STZ-induced hypoinsulinemia or alteration in HDL, SGOT, SGPT, serum total protein, urine total protein and potassium levels, it significantly reduced serum glucose, cholesterol, triglyceride, VLDL and LDL levels in STZ-diabetic rats. Upon treatment, significant increase in serum albumin level, urine output, urinary creatinine level and creatinine clearance, while decrease in sodium retention, serum urea, creatinine and urinary albumin levels was observed. Histopathological study of kidney showed 25% protection in mesangial cell proliferation as compared to diabetic control rats, upon treatment with Aqueous Extract, while hyalinization of renal tubules remained unaffected. Thus treatment with Aqueous Extract (levodopa concentration measured in our study was 3-3.5%) restored glucose and lipid metabolism. In conclusion, *Vicia faba* appears to be beneficial in diabetes mellitus and associated diabetic lipid and kidney related complications by its antihyperglycemic effect, antihyperlipedemic effect and protective effect on renal function by which may be because of high levodopa content present in the plant.

**536**

**A partially purified antineoplastic protein from Indian black scorpion (*Heterometrus bengalensis* koch) venom active against human leukemic U937 and K562 cells**

Das Gupta S, Vedasiromoni JR, Gomes A

**Indian Institute of Chemical Biology, Kolkata, India.**

**Introduction:**

Venoms are natural treasure trove of various bioactive molecules. Scorpion venom has been used in the tradition medicinal practices of Cuba, China and India from times immemorial. The present study aims to establish the antineoplastic activity of a partially purified protein, obtained from Indian black scorpion venom against human leukemic U937 and K562 cells.

**Methods:**

Crude venom was extracted from live adult scorpions by electrical stimulation to the telson. Venom was subjected to ion exchange chromatography using increasing molarities of NaCl in phosphate buffer. Venom fraction eluted with 0.5M NaCl showed cytotoxic activities against human leukemic cells and was named Hbv2. Antiproliferative activity was assayed by cell growth inhibition studies, fluorescence and confocal micrography. Apoptosis and stage of cell cycle arrest was evaluated by flow cytometer. Bcl2, Bax and caspase (3 and 9) levels in treated cell were measured.

**Results and Conclusion:**

Both U937 and K562 cell growth was inhibited by Hbv2 as evidenced by trypan blue exclusion method. Fluorescence micrography revealed damaged cell membrane and disintegrated nucleus due to Hbv2 treatment. Confocal micrographs showed severely damaged nuclear material in the treated cells. Flow cytometric analyses showed the presence of apoptotic cells with cell cycle arrest at G_0_ stage. Caspase 3 and 9 levels were augmented followed by an upregulation of proapoptotic bax and decrease of antiapoptotic bcl2. Thus Hbv2 showed cytotoxic activities against human leukemic U937 and K562 cells and the cell death might be brought about in an apoptotic manner.

**537**

**L-type calcium channel activity and its sequencing information in goat ruminal artery**

Pallai S^1^, Sahoo PK^2^, Mohanty BR^2^, Parija SC^1^

**^1^Orissa University of Agriculture and Technology, Bhubaneswar, India, ^2^Central Institute of Fisheries and Aquaculture, Bhubaneswar, India.**

The effects of three Ca^2+^ Channel blockers (CCBs), diltiazem, nifedipine and amlodipine on CaCl_2_-, KCl- and noradrenaline (NA)-induced contractions were examined to evaluate the relative sensitivity of L-type calcium channels in isolated goat (*Capra hircus*) ruminal artery (GRA). The ruminal artery ring (2× 2.5 mm) was mounted into a four chambered automatic organo-bath (PanLab) with the help of a L-shaped ss-hook connected to isometric force transducer (MLT 0201, Power Lab). The contractile tension was measured by Powerlab 8/32 with the help of lab chart software (AD Instruments, Australia). The relationship between the effects of Ca^2+^ entry blockers and the extracellular Ca^2+^ dependence of the contractions was also examined. In ‘nominally’ Ca^2+^ -free medium, addition of CaCl_2_ induced concentration-dependent contractions of previously depolarized arteries exhibited sensitivity in the order of nifedipine > amlodipine> diltiazem. The Ca^2+^ entry blockers induced concentration-dependent relaxation of the pre-contracted arteries (100 mM KC1) with the following order of potency: nifedipine is about 20 fold greater than amlodipine and is about 70 fold greater than diltiazem. Similarly, NA (10μM)–induced sustained contraction was inhibited by these CCBs in a concentration dependent manner in the order of potency i.e. diltiazem> amlodipine> nifedipine. Dihydropyridine (DHP)–sensitive Ca^2+^ agonist Bay k8644 (1ηM- 10μM).induced concentration related response with mean EC_50_ 0.104μM. The functional study clearly demonstrated that GRA exhibited a low degree of sensitivity to CCBs which may be due to expression of low affinity L-type Ca^2+^ channels in this vascular tissue. Further, L-type Ca^2+^ channel gene was amplified from RNA extracted from GRA by RT-PCR using self-designed primers. A 413 bp amplicon was produced in RT-PCR and the PCR product was sequenced. On analysis a nucleotide sequence identity of 83-93% was obtained when compared with other closely related mammalian sequences, taking into consideration of 382bp sequence product. Using BioEdit Sequence Alignment Editor, the goat calcium channel amino acid sequence was aligned with published calcium channel sequence of other published species, highly conserved blocks of amino acids are found in all species examined. In view of the functional study the reduced sensitivity of L-type Ca2^+^ channel in GRA could be due to variation in their sequence identity.

**538**

**Synthesis and evaluation of cytotoxicity of imidazolidinone analogues of aminoflavone**

Sudheer M^1^, Srinivasan KK^1^, Gopalankutty N^1^, Naseer M^2^

**^1^Manipal College of Pharmaceutical Sciences, Manipal, India, ^2^Indian Institute of Science, Bangalore, India.**

The flavonoids were proved as potential pharmacophores and are comparably safer molecules. The present work involves the synthesis of imidazolidinone analogues of flavonoids and evaluating its cytotoxic and apoptotic potentials. The imidazolidinones are an important group of compounds possessing various biologic activities. In the present research work, the 6-amino flavone and 6-amino 3-methoxy flavones were synthesized. These were then condensed with various oxazolidinones to obtain the novel proposed compounds, the 6-imidazolidinone substituted flavones. The newly synthesized imidazolidinone analogues were screened for in-vitro cytotoxicity on human cell lines by the MTT assay and SRB assay. Cervical cancer cell line, HeLa is used for the study. After confirming the cycyotoxic potential of the newly synthesized compounds, they were further characterized for their apoptotic potential by the cell cycle analysis and Hoechst staining. The results obtained were promising. The parent 6-aminoflavone showed a cytotoxicity in both MTT and SRB methods with an IC_50_ of 65±23.5*µ*g/ml and the parent 6-amino 3-methoxy flavone with an of IC_50_ 38±19*µ*g/ ml. The Imidazolidinone analogues obtained from these parent molecules showed an enhanced cytotoxicity with the highly potent derivative showing an IC_50_ of 1.78±35*µ*g/ml. Five highly cytotoxic compounds whose IC_50_ values are in the range of 1 *µ*g/ml to 10 *µ*g/ ml were selected from the newly synthesized compounds. HeLa cells treated with compounds for 48 hours shows condensed nuclei, DNA fragmentation as revealed from Hoechst staining and fluorescent microscopy and shows increased percentage of cells in sub G_0_ phase and disturbance in normal cell cycle. Cells treated with compounds shows apoptosis comparable with doxorubicin. The study had resulted in new molecules showing the apoptotic potential.

**539**

**Cytotoxic activity of flavanone from the stem of *Bauhinia variegata* linn**

Manan JK, Anil OK, Rajkapoor B, Kumar EP

**St John’s Pharmacy College, Bangalore, India.**

A flavanone has been isolated first time from the stem of *Bauhinia variegata*, and its structure was identified by colour reactions and spectral analysis. In a search for novel anticancer compound from medicinal plants, the isolated flavanone was tested for cytotoxic activity against 57 human tumour lines representing leukemia, nonsmall cell lung, colon, central nervous system, melanoma, ovarian, renal, prostate and breast cancers by using sulforhodamine B (SRB) assay method. The compound exhibited good cytotoxic activity with average 50% growth inhibition in the range of 4.06–4.81 *µ*M. The compound was more selective against OVCAR-5 (4.0 *µ*M) and SKOV-3 (4.06 *µ*M) cell lines of ovarian cancer. From these results it can be concluded that the compound isolated from *Bauhinia variegata* is selectively toxic against human tumour cell lines.

**540**

**Evaluation of antidepressant activity of aqueous extract of *Bacopa monniera* and its possible mechanism of action**

Niphadkar PV, Bhaskar M, Jagtap AG

**Bombay College of Pharmacy, Mumbai, India.**

**Introduction:**

Pain and depression share common neurochemical mechanisms. It has been suggested that use of an antidepressant with analgesic activity would help the patients with persistent pain in preventing relapse and achieving total symptom remission. A methanolic extract of *Bacopa monniera* has been reported to have antidepressant activity. Present study investigates the antidepressant and antinociceptive activity of Aqueous Extract of *Bacopa monniera* (AEBM).

**Methods:**

The antidepressant effect of AEBM (20 and 40 mg/kg p. o) was evaluated using swim and tail suspension test in mice. The analgesic effect of AEBM (80, 120 and 160 mg/kg p.o) was evaluated using supraspinal (hot-plate), spinal (tail-immersion) and peripheral (acetic acid induced abdominal writhing and formalin) methods.

**Results:**

The antidepressant effect of AEBM was found to be dose-dependent and significant when compared to Fluoxetine (20mg/kg, i.p). The analgesic effect of AEBM was compared with synthetic antidepressants and analgesics like Amitriptyline (10mg/ kg, i.p), Nortriptyline (20mg/kg, i.p), Tramadol (25mg/kg, i.p) and Aspirin (100mg/kg, p.o) using above mentioned methods. AEBM produced significant and dose-dependent antinociceptive effect in hot plate and formalin methods, while AEBM (120 and 160 mg/kg) produced significant and dose dependent antinociceptive effect in tail-immersion and acetic-acid induced abdominal writhing methods. Administration of beta-1 antagonist Atenolol (1 mg/kg. i.p) and alpha-2 antagonist Yohimbine (1 mg/kg, i.p) inhibited antinociceptive effect induced by AEBM (80, 120 and 160mg/kg, p.o) whereas Naloxone (2 mg/kg, i.p) partially inhibited antinociceptive effect induced by AEBM (80, 120 and 160mg/kg, p.o).

**Conclusion:**

Our results indicate that *Bacopa monniera* demonstrates antidepressant as well as peripheral and central antinociceptive effect. This data also suggests that *Bacopa monniera* induced antinociception involves adrenergic and opioidergic pathways.

**541**

***In vitro* antioxidant property of some selected fruits of north east India: A comparative study with other widely used citrus fruits**

Mudoi T^1^, Devi R^1^, Deka DC^2^

**^1^Institute of Advanced Study in Science and Technology, Paschim Boragaon, Guwahati, ^2^Gauhati University, Guwahati, India.**

*In vitro* antioxidant properties of *Citrus grandis* and *Garcinia pedunculata* have been studied, and these have been compared with some of the widely used citrus fruits such as Mouchumbi, Lime and Orange. *Citrus grandis* and *Garcinia pedunculata* are two uncommon local fruits available in Northeast region of India, but their commercial potentialities are yet to be properly explored. We are trying to explore the importance of these fruits in terms of food value as well as commercial exploitation. Experimental studies on the antioxidant properties of these fruits indicate that these fruits possess very high antioxidant property as compared to many other widely used citrus fruits. While *Citrus grandis* and *Garcinia pedunculata* have antioxidant property as high as 93% and 92% respectively, Mouchumbi, Lime, and Orange, on the other hand, have antioxidant property 28.37%, 45.48% and 85.18% respectively. Trolox equivalents of *Citrus grandis* and *Garcinia pedunculata* are 0.0489mM and 0.0485mM respectively and those of Lime, Mouchumbi and orange are 0.0149mM, 0.0239mM and 0.0447mM respectively. From these studies it is very clear that *Citrus grandis* and *Garcinia pedunculata* do possess significant food value, and their commercial exploitation should be highly viable. This paper will report the methodology of our study and the results in detail. Abundance of trace metals in these fruits as micronutrients will also be reported.

**542**

**Peak expiratory flow rate of medical students**

Malhotra Varun, Dhungel Kshitiz Uppadhay , Patil Rajkumar , Shankar Ravi, Bhaskhar Vijay , Sen Tarun K, Binu VS, Dhawan Vikram , Malhotra Monica , Mallikarjuna PT, Srinivasaragavan N, Rajesh

**Vinayaka Missions Medical College, Salem, Tamil Nadu, Manipal College of Medical Sciences, Nepal**

Peak expiratory flow rate (PEFR) is the maximum rate of airflow achieved during a forced expiration after maximal inspiration. PEFR of 59 healthy medical students was measured with FERRARIS Pocket flow meter. After proper rest the subjects were requested to take deep breath and exhale as forcefully as possible in one single blow into the instrument. During the breathing the subject’s nose was closed and a tight seal was maintained between lips and mouthpiece. PEFR is recorded to the nearest l/min. The height in centimeters and weight in kilograms is recorded. Pearson’s correlation coefficient is performed to examine the relationship between PEFR, height, weight and Body Mass Index (BMI) using SPSS (version 10.0). The mean height, weight, BMI and PEFR is 5.5 ± 0.3 feet, 60.1 ± 10.5 kg, 22.2 ± 4.1 kg/m^2^ and 454.3 ± 64.5 L/min respectively. There is an increase in “flow rates” with increase in height, weight and BMI. Body mass index is an important parameter to assess whether the subjects are obese is significantly correlated with PEFR. Physical activity reduces weight and improves fat distribution in the body. Therefore, it can be predicted that physical activity improves PEFR. The more the height and weight, more will be the oxygen demand for the tissues. The increased oxygen demand should be met by more ventilation thus, increasing respiratory function and hence PEFR.

**543**

**Quality control in dope testing of threshold substances: Role of measurement uncertainty-– a case study**

Kaur T, Dubey S, Jain S, Beotra A

**National Dope Testing Laboratopry, New Delhi - 110003, India.**

**Introduction:**

The use of approximately 55 stimulants in sports is prohibited by World Anti Doping Agency (WADA) For analysis of Cathine, a threshold limit of 5 ug/ml has been set up as this is available in cough and cold preparations. For the quantitation of threshold substances, estimation of Measurement Uncertainty (MU) is a requisite as described in WADA International Standard for Laboratories One of the Proficiency testing (PT) sample containing Cathine was received from WADA. It was sent to 34 WADA accreditated labs in the world including NDTL which was in probationary phase of WADA accreditation at that time. The result reported by NDTL after consideration of MU did not fall within acceptable range (i.e. Z score was more than 3). Hence, the aim of this study was to find out the root cause of higher value of cathine in one of the WADA PT sample.

**Materials and Methods:**

Series of experiments were conducted to rule out various parameters which could contribute to uncertainty like weighing balance, micropipette, solvent used, standard used (internal and, reference standard) etc. Drug free urine samples were spiked at two concentrations i.e. 5 and 8 ug/ml and processed in duplicate and injected on Agilent 6890 GC-NPD for analysis and quantitation.

**Result:**

The higher values of Cathine were found to be very consistent even after making changes in various parameters except freshly procured reference standard, thereby showing reproducibility and robustness of the method. When freshly procured reference was used, the value of Cathine was found within permissible range. Hence, the higher values were due to deterioration of reference standard which was purchased earlier on which no expiry date was mentioned.

**Conclusion:**

It is concluded to formulate and implement a policy with regard to the validation criteria for stability of reference standards of threshold substances even without expiry date.

**544**

**Evaluation of antistress, antianxiety and hypnotic activity of a polyherbal formulation**

Gupta AVSSS, Bharathi K N, Sivaramaiah N

**Visveswarapura Institute of Pharmaceutical Sciences, Bangalore, India.**

**Introduction:**

The pr esent study was undertaken to investigate the Antistress activity, Antianxiety activity and hypnotic activity of VEDIC CALM comprising of Bacopa monnieri, Centella asiatica and extracts of other related plants.

**Methods:**

In Antistress activity Chronic stress was induced by cold immobilization for 10 days in rats. The degree of protection in treated animals was determined by measuring gastric ulceration, adrenals, spleen, liver weights, serum glucose, AST, ALT, cholesterol and W.B.C count. Withania somnifera was used as standard. In Antianxiety activity the number of entries and time spent in open and closed arms of Elevated Plus Maze, were observed in treated rats and compared with standard and control. The numberof entries and time spent in light box of light and dark box were observed in acute study. Diazepam was used as standard. In hypnotic activity the potentiation of thiopental induced sleeping time was evaluated for polyherbal formulation in mice.

**Results:**

Vedic calm and Withania somnifera pretreatment in rats significantly reversed all the changes those were due to stress such as occurrence of gastric ulcer, elevation of adrenals weight, liver weight, serum glucose, AST, ALT, cholesterol, W.B.C and reduction of spleen weight. The Vedic calm and diazepam significantly increased the time spent and number of entries into open arms and light box in elevated plus maze and light and dark box model respectively. The Vedic calm significantly potentiated onset and duration of thiopental induced sleeping time in mice.

**Conclusion:**

The Vedic calm showed significant antistress, antianxiety and hypnotic activity.

**545**

**Antinociceptive activity of ethanolic extract of bark of *Cassia fistula***

Sakhare GR^1^, Vadnal PY^1^, Patwardhan SK^1^, Singhai AK^2^, Somani RS^1^

**^1^Sinhgad College of Pharmacy, Pune, India, ^2^Dept. of Pharm. Sci., Dr. HS Gour University, Sagar, India.**

**Objective:**

Aim of the present study was to evaluate antinociceptive activity of ethanolic extract of bark of *Cassia fistula* (Family: Leguminoseae).

**Method:**

The ethanolic (95%) extract of bark of *Cassia fistula* (EECF) was prepared by maceration. EECF was examined for analgesic activity by using hot plate, formalin induced paw licking and acetic acid induced writhing method. The doses 200 mg/kg and 400 mg/kg were administered orally. The results of EECF treatment were compared with control group.

**Result:**

The results obtained showed that the treatment with EECF (200 and 400 mg/kg, p.o.) significantly increased pain threshold in hot plate method, produced a marked inhibition of the nociceptive response in both neurogenic and inflammatory phases of formalin test and decreased number of writhing in acetic acid induced nociception as compared to control group animals.

**Conclusion:**

*Cassia fistula* possesses both central as well as peripheral analgesic activity.

**546**

**Impact of arsenic poisoning**

Datta BK, Mandal P, Dey GN, Rana T, Ghosh C, Sarkar S, Bera AK, Bhattacharya D, Chakraborty AK, Mandal TK

**Department of Pharmacology and Toxicology, West Bengal University of Animal and Fishery Sciences, Mohapur Campus,P.O.-Krishiviswavidyalaya, Dist. Nadia, India.**

**Objective:**

To explore the source of Arsenic toxicity in cattle of arsenic prone area of West Bengal and also to identify the food chain of arsenic exposure in human being.

**Materials and Methods:**

Eighty milch cattle from three villages were selected. Sixty animals from two villages of arsenic affected area and twenty from nonarsenic affected area which was considered as control zone. Hair, straw, drinking water, feces, milk and urine samples from each animal were estimated by Atomic Absorption Spectrophotometer using hydride generation technique (Varian AAS coupled with vapour generation system). Recovery percentage of each substrate varied from 86 to 94%.

**Results and Discussion:**

Hair and feces contained high amount of arsenic in cattle of arsenic prone area. Significant amount of arsenic could be detected in urine sample while level of arsenic in milk of cattle was above MRL. Arsenic level in milk of cattle reared by the family affected with arsenic toxicosis was higher compared to non affected family. Straw samples contained high amount of arsenic in test animals of arsenic prone villages compared to drinking water samples.

**Conclusion:**

Arsenic may accumulated in cattle of arsenic prone villages via feeding of straw rather than water. Major source of arsenic contamination in cattle of arsenic prone villages may be through feeding of straw rather than drinking water.

**547**

**A novel lead (Liv-1) from natural source that improve the quality of life in varying ailments**

Jaggi B S, Suri KA, Gupta B D

**Indian Institute of Integrative Medicine, Canal Road, Jammu, India.**

A single herbal formulation has been developed after years of dedicated work, based on the preclinical studies on chemically standardized extract of *Vitex negundo* Linn. This led to the development of a single herb formulation (Liv-1), a hepatoprotective against viral and alcoholic liver disorders. Liv-1 is standardized based on two identified chemical markers and has been licensed to M / S Medley pharmaceutical Ltd, Mumbai. The company after conducting the clinical trials as proof of efficacy and safety studies has finally launched the product as standardized ayurvedic herbal drug for hepatic disorders both in tablets and syrup form. Under preclinical (*in vivo* and *in vitro*) studies it has been found to be efficacious against hepatotxins and the optimum dose has been found to be 25 mg/kg, p.o. while the ED_50_ has been 25 ± 5.0 mg/ kg, and p.o. The product has been found to be more efficacious than other commercially available herbal formulation viz. Silymarin, Liv-52, Tefroli, Hepartoguard and Stmuliv. The oral LD_50_ in rats was found to be > 2g/kg, (n=10) Sub-chronic toxicity, teratogenecity and stability studies have established that Liv-1 is extremely safe with a therapeutic index of more than 60. Human dose has been calculated to be 2.5 to 3 mg/kg, (B. D).This study validates the use of *Vitex negundo* Linn., as a remedy for liver disorders, in Indian traditional system of medicines.

**548**

**Infuence of some antihyperglycemics on wound healing in euglycemic male Wistar rats**

Ambrish C, Torgal SS, Patil PA

**J.N.Medical College, Belgaum, India.**

**Introduction:**

Wound being one of the common clinical entities, often challenges the clinicians when associated with diabetes mellitus. Antihyperglycemics like metformin has been reported to increase angiogenesis through activation of AMP-activated protein kinase and promote wound healing in diabetics. There are controversial reports regarding rosiglitazone on angiogenesis in diabetics and no study has been reported the effect of acarbose on wound healing. Prohealing activity, if any of these drugs could be exploited to treat non-diabetic wounds. Therefore, the present study is planned to investigate the effect of these drugs on wound healing of different types of wounds in euglycemic Wister rats.

**Methods:**

Inclusion criteria - Rats with blood glucose level within normal range. Rats were divided into control and drug treated groups. Under light ether anaesthesia animals were inflicted with resutured incision, excision and dead space wounds. Clinically equivalent doses of drugs were given orally OD for 10 days in resutured incision and dead space wounds, while treatment continued till complete epithelisation in excision wound groups.

**Results:**

Metformin and acarbose significantly enhanced healing in all the wound models as compared to controls, while rosiglitazone failed to do so in any of the wound models. Hydroxyproline content and histological studies of the granulation tissue confirmed the influence of above mentioned drugs on wound healing.

**Conclusion:**

The pro-healing property of metformin and acarbose needs to be confirmed clinically.

**549**

**Synthesis and characterization of methotrexate analogues for anticancer activity**

A Prakash^1^, A Shayam Sundar^2^, Suresh Kumar K^2^, Ganguly Swastika^3^

**^1^IHBAS, Delhi, India, ^2^KMCHCOP, Coimbatore, India, ^3^BIT, Misra, Ranchi, India.**

Research and development of Methotrexate for cancer chemotherapy have been one of the major focus in drug design. Unfortunately, several problems are associated with clinical use of methotrexate such as resistance and high level of toxicity to bone marrow and gastrointestinal mucosa. Therefore the present work is aimed to synthesize new analogues of methotrexate targeting pteridine nucleus.

**Methods:**

1. Synthesis of methotrexate analogues
2. Characterization of synthesized analogues IR Spectra and Mass Spectra
3. Screening of biological activity *In vitro* activity in DLA cell line *In vivo* activity in DLA tumor cell induced Swiss albino female mice.

**Results:**

The synthesized compound A and B were characterized and confirmed for new entity of methotrexate analogues by physiochemical and spectral studies. The reliable criteria for judging the activity of an anticancer drug *in in-vivo* is the body weight analysis, mean survival time and the prolongation of the life span of animals. The test compounds were found to be safe up to a dose of 5mg/kg body weight on oral administration in experimental animals and were found to be effective in inhibiting cancer growth by both *in vitro* and *in vivo* screening.

**Conclusion:**

The results confirmed that compound B was found to possess more potent anticancer activity by decrease in body weight and prolongation of life span than compound A. It could be concluded, substituting methyl quinazoline nucleus at the 4^th^ position of pteridine nucleus in methotrexate molecule, a potent analogue with significant antitumor activity can be synthesized with ease. It is only a preliminary review of methotrexate analogues, which can be subjected for future evaluation.

**550**

**A study on drug prescribing pattern in Meerut city**

Sharma M^1^, Gaur N^2^

**^1^LLRM Medical College, Meerut, ^2^Subharti Medical College, Meerut, India.**

**Introduction:**

Medical audit improves the standards of medical treatment. The study of prescribing pattern is a component of medical audit which seeks monitoring, evaluation and necessary modifications in the prescribing practices of the prescribers to achieve rational and cost effective medical care.

**Materials and Methods:**

Prescription data was collected prospectively over a 6-month period from Government and private hospitals, and private practitioners of Meerut city. All relevant information was entered into a preformed proforma and the prescriptions were analysed under the following parameters as recommended by WHO: 1.average number of drugs per encounter; 2.percentage of drugs prescribed by generic names; 3.percentage of encounters with antimicrobials; 4.percentage of encounters with injectables; 5.commonly prescribed drugs; 6.percentage of drugs prescribed from National essential drugs list.

**Results:**

The average number of drugs per encounter were 3.8. 9.2% drugs were prescribed by generic names. The percentage of encounters with antimicrobials and injectables were 45.7% and 16.3%, respectively. The commonly prescribed drugs included NSAIDs(32%), antimicrobials(23.8%), antiulcer drugs(11.2%). Percentage of drugs prescribed from National essential drugs list was 18.8%.

**Conclusions:**

This study provides baseline data for monitoring future prescribing trends. A tendency towards prescribing by brand names was observed, probably based on the confidence of prescribers in a particular brand. Nevertheless, prescribing by generic names should be encouraged to reduce the cost of drug treatment to patients. To promote prescribing of drugs from National essential drugs list, awareness should be generated about WHO program on Rational Use of Drugs.

**551**

**Study of crude *Bungarus caereleus* snake venom (BCV) on macrophage**

Bhattacharya S, Das T, Vedasiromoni JR, Gomes A

**Indian Institute of Chemical Biology, Kolkata, India.**

**Introduction:**

Macrophage is an important armor of immune system, and when it gets activated it spreads for phagocytosis, and/ or it releases various cytokines that may have beneficial effects like tumoricidal and bactericidal activities. On the other hand snake venom is a natural biological resource, which contains several components of potential therapeutic values. Keeping these in mind, our present study was aimed to find a correlation between crude *Bungarus caereleus* venom with macrophage activation, and, if the macrophages get activated by crude BCV it may boost immune system.

**Methods:**

Venom was collected from live *Bungarus caereleus* snakes and was lyophilized. Peritoneal macrophages were obtained from BalbC mice by intra peritoneal injection of 4% starch solution. Ehrlich Ascites Carcinoma (EAC) cells were intraperitoneally inoculated in BalbC mice. Nitric Oxide (NO) synthesis was measured by micro plate assay method using Griess reagent.

**Results and conclusion:**

From the above study it was found that BCV elevated number of macrophages along with its spreading in venom treated mice in 24 hrs as compared to control. NO production also increased in comparison to control. In EAC inoculated mice the venom also increased macrophage count and spreading after 3 days of pretreatment. Earlier work from this laboratory found that 10 days pretreatment with BCV on EAC inoculated mice inhibited 56% of EAC cell count. BCV induced macrophage activation may lead to increased levels of cytokines that may partially affect EAC cell growth. Further parameters like TNF-α, IL-1, and IL-6 will be evaluated in normal, EAC and venom treated mice.

**552**

**Drug utilization pattern of idiopathic childhood epilepsy in tertiary health care center**

Begum S, Biswas DK, Ghosh S, Maiti SP, Mondal S

**R.G. Kar Medical College, Kolkata, India.**

**Introduction:**

The study was conducted to know the prevalence of various types of Idiopathic Childhood Epilepsy and the utilization pattern of Antiepileptic drugs (AED) in the tertiary referral centre in R.G Kar Medical College and to compare the efficacy and safety profile of commonly used AED.

**Methods:**

A total of 100 Idiopathic epileptic patients of both sexes below 12 yrs of age who were prescribed an AED were considered for analysis. Demographic profile, type of epilepsy, AED, number of epileptic events, biochemical, EEG and ADR data were collected and analyzed.

**Results:**

78% of epileptic children belonged to age group of 5 to 12 year, 56% were male and 89% were underweight. Incidence of Generalized Tonic-Clonic Seizure (GTCS) was 80%, Complex Partial Seizure 60%, Partial Seizure with Secondary Generalization 32%, Simple Partial Seizure 8%, Myoclonic 8%, Absence 6.67% and Atonic 3.33%. 97% of epileptic children received monotherapy. Valproate was the most commonly prescribed drug in GTCS, Atonic, Myoclonic and Absence seizure. Carbamazepine was commonly prescribed drug in Partial seizure. Carbamazepine produced side effect (SE) in 85.29% and both Phenytoin and Valproate produced SE in 73% of patients. It was observed that to achieve fastest seizure control in 95% of patients, Valproate, Carbamazepine and Phenytoin required 13.92%, 33.24%and 19.62% of monthly family income respectively.

**Conclusion:**

Idiopathic Childhood Epilepsy is more common in underweight male children. GTCS is the commonest type of epilepsy. The majority of epileptic children receive monotherapy. Valproate is the commonly prescribed drug in all type of epilepsy other than Partial seizure. It is the most cost effective and safest AED.

**553**

**A study of pattern of prescribing antibiotics prophylactically in vaginal deliveries and caesarian sections by the obstetricians of a north Indian referral hospital**

Bhanwra S, Gautam CS, Huria A

**Government Medical College and Hospital, Chandigarh, India.**

Prophylactic use of antibiotics in caesarian sections (CS) and in high risk vaginal deliveries is done commonly and has been shown to decrease the morbidity also.The present study was conducted to analyse the pattern of prescribing antibiotics prophylactically in CS and in vaginal delivery. The study was conducted retrospectively in a north Indian referral hospital by collecting data from the case records of 200 patients of vaginal deliveries and CS over a period of six months from the medical records department of the hospital. Out of all the vaginal delivery patients 60% were prescribed ampicillin alone and 20% of patients received a combination of ampicillin, gentamicin and metronidazole for a period of at least five days. 58% of CS patients received a combination of ampicillin, gentamicin and metronidazole while 30% were given cefazolin, gentamicin and metronidazole for five days. Only 5% of patients were given cefazolin alone and 7% were given ampicillin alone. All the drugs were started at least half an hour before going for the procedure (vaginal delivery or CS). All the antibiotics prescribed were generic drugs and were from the essential drug list except one. But they were overprescribed as per the guidelines of cochrane database of systematic reviews in both vaginal deliveries and CS. Further, amoxycillin would have been a better choice as regards bioavailability, safety and compliance in comparison to ampicillin. Timing of administering the antibiotic was also not appropriate, because it has to be administered after the cord clamping, as per the guidelines.

**554**

**Synthesis and *in vitro* antitumor activity of novel pyrimidine derivatives of benzothiazoles**

Manjula SN^1^, Arvind MB^2^, Vipan PK^1^, Vijay R^1^, Gopalan KN^1^, Mallikarjuna RC^1^

**^1^Manipal College of Pharmaceutical Sciences, Manipal, India, ^2^Sri Dhanvantri Pharmaceutical Analysis and Research center, Surat, India.**

**Introduction:**

The derivatives of benzothiazoles as well as pyrimidines have been reported as potent bioactive molecules. The present study reveals the synthesis and *in vitro* antitumor activity of novel pyrimidine derivatives of benzothiazoles.

**Methods:**

The acetyl derivative of 2-aminobenzothiazole was synthesized and treated with various substituted aldehydes at alkaline condition to obtain the styryl carbonyl pharmacophore. These compounds were cyclised in presence of thiourea/sodium hydroxide to yield the title compounds. QSAR studies for P^56^lck activity were analyzed by MOE software. The cytotoxicity of the compounds was assessed in two human cancer cells by MTT assay. The most promising compound was tested against various cancer cell lines at different time intervals by MTT assay. The IC_50_ values of the compounds were calculated. Further the cloning efficiency and cell cycle specificity of the most promising compound in MCF-7 cells were assessed.

**Results:**

All synthesized compounds were characterized by IR, ^1^H NMR,^13^C NMR, and expected spectra were obtained. Predicted IC_50_ values for P^56^lck were found significantly lower than standard. In the preliminary cytotoxicity test, one of the compounds was found potent with an IC_50_ value of 18.73 micromoles and showed sensitivity in other tested cell lines. Cell cycle analysis results indicated that the compound has no specific action on cell cycle.

**Conclusion:**

One out of ten synthesized compounds showed significant cytotoxicity in human cancer cells. The expected molecular mechanism may be the inhibition of P^56^lck, which is concluded by QSAR studies. The detailed studies are in progress to elucidate its molecular mechanism.

**555**

**Korean ginseng extract attenuates reserpineinduced orofacial dyskinesia and improves cognitive dysfunction**

Kharjul MD, Sanghavi CR, Mohan M, Kasture SB

**MGV’s Pharmacy College, Nashik 422 003, India.**

**Introduction:**

Reserpine induced orofacial dyskinesia, commonly used as a putative model for TD, is an involuntary movement; most commonly affecting the orofacial region (i.e.buccolingualmasticatory dyskinesia). We have studied effect of K. ginseng extract on this model.

**Materials and Methods:**

*Korean ginseng* extract (KGE, 100 or 200 mg/kg) suspended in distilled water was administered orally for 21 days, with or without reserpine (1mg/kg s.c.) administered on day (1, 3, and 5). One groups received only vehicle. Animals were divided in 3 groups, which received vehicle. The vacuous chewing movements (VCM), orofacial bursts and tongue protrusions (TP) were measured in each rat on day 7, 14, and 22. Since reserpine treated animals show poor retention of memory in the elevated plus maze paradigm, the effect of KGE was also studied on cognition. Antioxidant enzyme estimations (SOD, CAT and GSH) of forebrain were carried on day 22 after completion of behavioral assessment.

**Results:**

Chronic administration of KGE significantly (*P* < 0.05) inhibited reserpine induced VCMs, orofacial bursts, and TP. Chronic *Korean ginseng* (200 mg/kg) administration significantly reversed reserpine-induced retention deficits. KGE significantly increased level of Superoxide dismutase (SOD), Catalase (CAT), Glutathione (GSH), and reversed the effect on lipid peroxidation compared with reserpine group.

**Conclusion:**

The present study concludes that oxidative stress might play an important role in reserpine-induced abnormal oral movements. *Korean ginseng* extract strengthens defense mechanism and attenuates reserpine induced orofacial dyskinesia and improves memory.

**556**

**A study of the antihistamine drug use in the ENT ward of a tertiary care teaching hospital**

Braich JS, Singh J, Wadhera R, Gulia JS, Gulati SP

**PGIMS, Rohtak (Haryana), India.**

**Objective:**

To study the prescribing pattern of antihistamines (H_1_ blockers) in operated patients (MRM, FESS, septoplasty, myringoplasty and tonsillectomy) of ENT Department of PGIMS, Rohtak.

**Methods:**

Prescribing pattern was evaluated by collecting the data of indoor patients of the department over a period of three months. Relevant information was entered in a preformed proforma and analyzed for various parameters.

**Results:**

Total 497 prescriptions were analyzed. Total drugs prescribed were 2504 which included oral 1466(58.54%), parenteral 826(32.98%), and topical 212(8.46%). Average number of drugs per prescription was 5.04. The fixed dose combinations (FDC) used were 693 (27.67%) and the essential drugs used were 865 (34.54%). Antimicrobial agents (AMA) prescribed were 857(34.22%), analgesics were 385(15.37%) and antihistamines were 405 (16.17%). Fixed dose combination, nimes ulide+pseudoephedrine+cetirizine (Namcold), was maximally used among antihistamines i.e. 98 (24.19%) followed by levocetirizine+ph enylpropanolamine (62), cetirizine (51), cetirizine+pseudoephedrine (42), levocetirizine+ambroxol (35), promethazine (32), levocetirizine (29), chlorpheniramine (9), ibuprofen+pseudoephedrine+chlorphe niramine (17), loratadine (15), mezolastine (13), chlorpheniramine (9), and ebastine (2). Total antihistamine fixed dose combinations used were 219(54.07%).

**Conclusion:**

Various antihistamines have been used in the post operative patients of the ENT ward of PGIMS, Rohtak; and the use of antihistamines as fixed dose combinations has been found to more common.

**557**

**The website as a tool for reporting poor quality of drugs supplied to a hospital: The JIPMER experience**

Gitanjali B

**JIPMER, Pondicherry, India.**

The quality of medicines being dispensed to patients availing government health facilities in many developing countries has been documented to be poor. In JIPMER, Pondicherry, a 1050 bedded tertiary care hospital, it was found that the quality of drugs being supplied to the hospital was erratic, despite using the Delhi model of pooled procurement. This led to numerous complaints from patients and clinicians. In order to hold drug manufacturers responsible for their actions, it was decided to open a website on which quality issues regarding drugs supplied to the hospital would be put up, along with photographs and the action taken report. It was surmised that if the companies supplying the drugs knew that such information would be accessible to all, they would perhaps comply with the quality standards. In 2007, The World Health Organization, funded a project in JIPMER Pharmacy, and a website www.jipmer.edu/pharmacy added to the main website of the institute. Training was given to the pharmacists to take digital photographs and upload information regarding poor quality of drugs supplied, the details of the supplier and violation of tender norms. The website has since attracted a lot of attention among drug suppliers who are wary of supplying poor quality products since the information is available to all future clients. This initiative is a way of sharing information with other institutions across the country regarding the poor quality of drugs supplied by manufacturers and will hopefully guide other hospitals in their purchase.

**558**

**Study of antimicrobial susceptibility pattern of bacterial isolates from CSF in pediatric patients of septic meningitis in a tertiary care hospital**

Chugh Y, Sharma A, Kastury N, Kapoor AK, Bhargava A, Srivastava AK

**MLN Medical College, Allahabad, India.**

**Objective:**

To study antimicrobial susceptibility pattern of bacterial isolates from CSF in pediatric patients with clinically suspected septic meningitis admitted to SN Children Hospital, attached to MLN Medical College, Allahabad.

**Materials and Methods:**

Study was conducted from Aug 07 to July 08. All the patients aged upto 12 years admitted with clinically suspected septic meningitis in SN Children Hospital were included. CSF was collected with all aseptic precautions prior to administering antibiotics for culture and sensitivity using Kirby Bauer Disc Diffusion method following NCCLS guidelines.

**Results:**

Out of 396 cases studied, 86 cases were culture positive and remaining sterile. Of culture +ve cases, 66% were Gm +ve and 33% Gm +ve. Among these, Gm +ve organisms showed higher sensitivity to *teicoplanin, linezolid, vancomycin, meropenem, amikacin*, combination of *piperacillin+tazobactam*, moderate sensitivity to *gatifloxacin, gentamicin, netilmicin, ofloxacin*, combination of *cefoperazone+sulbactam, amoxicillin+clavulanic acid*, but resistance to *cephalosporins, erythromycin, cotrimoxazole, chloramphenicol, ampicillin, amoxycillin and penicillin-G*. While Gm +ve organisms showed higher sensitivity to *amikacin, aztreonam*, combination of *piperacillin+tazobactam*, moderate sensitivity to *ofloxacin, carbenicillin, meropenem, gatifloxacin*, combination of *cefoperazone+sulbactam, amoxicillin+clavulanic acid*, but resistance to ampicillin, cotrimoxazole, ciprofloxacin, norfloxacin and cephalosporins.

**Conclusion:**

Irrational and inappropriate use of antibiotics is responsible for development of resistance. To prevent this, there should be formation of effective national or state level antibiotic policy and guidelines. Government and other regulatory bodies should conduct regular surveillance programmes to monitor the antimicrobial susceptibility pattern area wise. Updated information regarding pattern should be imparted to clinicians by organizing continuous medical education (CME) programmes.

**559**

**Antitumor activity of some novel 2-quinolone derivatives in DLA induced solid tumor in swiss albino mice**

Randhawa N, Nitesh K, Thomas S, Dhamija I, Manjula SN, Vipan Parihar K, Jayashree BS, Mallikarjuna RC

**Manipal College of Pharmaceutical Sciences, Manipal, India.**

**Introduction:**

Farnesyltransferase inhibitors have emerged as a novel class of anti-cancer agents. Tipifarnib, a 3-Aryl-2-quinolone derivative acts as anticancer by similar mechanism and currently under clinical trials. Considering, 2-quinolone analogues are the powerful inhibitors of farnesyltransferase and novel targets for chemotherapy, a series of 2-quinolone analogues were synthesized in our lab and screened for antitumor activity.

**Methods:**

The standard MTT bioassay was used to screen all synthesized 2-quinolone compounds for *invitro* cytotoxicity in human adenocarcinoma cells (MCF-7) at 72 hours of drug incubation. Dalton’s lymphoma ascites cells (DLA) maintained and propagated ip by serial transplantation in adult Swiss albino mice were used to assess *invivo* cytotoxicity. Briefly, the known numbers of viable cells (1.0X106) were injected ip to the right hind limb of each mice and day was considered as zero. Drug treatment was started after 24 hours of tumor inoculation, and cisplatin (3.5mg/kg) was used as standard. Tumor weight and volume were assessed to determine the antitumor activity.

**Results:**

Among all synthesized compounds, JST 2 and JST13 were found maximum effective to cause cytotoxicity in MCF-7 cells, hence further explored in animal models. Both compounds also showed significantly antitumor activity in DLA inoculated mice. However JST2 at 200mg/kg was found most effective in inhibiting the tumor growth in DLA inoculated mice.

**Conclusion:**

The JST 2 and JST 13 possess significant antitumor and inhibit the growth of both cancerous cells (DLA and MCF-7).

**560**

**Role of MAPKs and oxidative stress in centchroman induced apoptosis**

Zaidi D, Nigam M, Ranjan V, Sharma R, Balapure AK

**Central Drug Research Institute, Lucknow, India.**

The antiestrogen Tamoxifen (TAM) is commonly used for the prevention and treatment of breast cancer. Centchroman (CC), another selective estrogen receptor modulator (SERM), has been shown to have potent antineoplastic action in MCF-7 (ER-ve) and MDA MB-231 (ER-ve) human breast cancer cells by us. It is undergoing Phase III Multicentric Clinical Trials in Stage III/IV breast cancer. We have also shown that CC mediates it’s antineoplasticity through apoptosis. Oxidative stress is a major factor contributing to drug induced apoptosis. We have explored it’s role in CC induced cell death by studying mitogen activated protein kinases (MAPKs), which are the principal signalling pathways involved in stress response. Using L-NAC as an antioxidant, we examined it’s effect on CC induced apoptosis using flow cytometry. The role of MAPKs was investigated by cytotoxicity assay (SRB) using MAPK inhibitors and by Western Blotting. Tamoxifen has been used as a standard positive control. Addition of ERK inhibitor in TAM treated cells increases apoptosis, whereas it has no effect on CC treated cells. In contrast, JNK and p38 inhibitors inhibit both TAM and CC induced apoptosis. These results are also supported by Western blotting which showed drug treatment induced up-regulation of phospho-p38 and phospho-c-Jun. Our studies with JNK and p38 inhibitors confirm the activation of these two pathways in TAM / CC induced apoptosis, implying the role of oxidative stress conclusively. CC has an added advantage over TAM that the ERK pathway, involved in proliferation, is not activated by it, improving the drug’s antiproliferative efficacy.

**561**

**Lercanidipine and orofacial pain: An experimental evaluation in rats**

Joshi R, Hota D, Mittal N, Chakrabarti A

**Department of Pharmacology, PGIMER, Chandigarh, India.**

The present study evaluated the efficacy of lercanidipine, a potent, long-acting, vascular-selective dihydropyridine calcium entry blocker in formalin induced orofacial pain in rats. Formalin injected into the vibrassal pad of rat produces grooming behaviour similar to human orofacial pain. Adult Wistar rats of either sex received an injection of 50 microlitres of 5% v/v subcutaneous formalin injection into one vibrissal pad and consequent facial grooming behaviour was monitored. Animals exhibited two distinct periods of nocifensive grooming: (i) an acute phase lasting 0-6 min; and (ii) a tonic phase lasting 6-45 min. Response of lercanidipine was noted at doses of 0.5, 1, 2.5, 5, 10, 25 micrograms/kg, i.p., administered 15 min prior to formalin injection. Lercanidipine produced dose dependent inhibition of facial grooming in both acute and tonic phases as compared to vehicle treated control group. The suppression of grooming response was significant at all dose levels as compared to vehicle. P-value in all the groups was found to be less than 0.01 except at 0.5 micrograms /kg in which *P*- value was < 0.05 in both acute as well as tonic phase. None of the animals exhibited any hypotensive episode or other ADRs during the study period suggesting high degrees of safety. Thus it may be concluded that, lercanidipine may be further evaluated either alone or as an adjunct for the treatment of various human facial and neuropathic pains.

**562**

**Evaluation of changes in prevalence and emergence of resistance among urinary isolates in a tertiary care hospital**

Kumar S, Yadav VS, Chugh Y, Sharma A, Shukla AK, Anand M, Kastury N, Tiwari AR

**MLN Medical College, Allahabad, India.**

**Objective:**

To evaluate and compare the prevalence and emergence of resistance among urinary isolates in clinically suspected patients of UTI attending SRN Hospital attached to MLN Medical College Allahabad.

**Materials and Methods:**

Urine culture and sensitivity data was collected from the Microbiology department from April to July 2008 and compared with that of April to July 2006.

**Results:**

[2008 vs 2006]: During both the years, approximately 1/3M^rd^ of clinically suspected patients (33.89%, 33.47%) were culture positive. During the year 2008 the commonest offending agent was *E.coli*(32%), followed by *Acinetobacter*(21%), *Citrobacter*(20%), *Klebsiella*(8%) and others(19%) whereas during 2006 the commonest was *E.coli*(51%) followed by *Citrobacter*(20%), *Klebsiella*(8%), *Acinetobacter*(7.5%) and others(16.5%). These 4 organisms constituted 80% of culture positive cases in both the years. During 2008 the prevalence of resistance among the commonly used antibiotics was highest with Ampicillin(87.25%), followed by Nalidixic acid(75%), Norfloxacin(75%) and Cephalosporins(67.6%), where as in 2006 the prevalence pattern was, Ampicillin(79.12%), Nalidixic acid (74%), Norfloxacin(66%) and Cephalosporins(56.5%). In both the years the lowest prevalence of resistance was reported with Cefoperazone-Sulbactum followed by Piperacillin-Tazobactum, Nitrofurantoin and Amikacin. Among them the rate of emergence of resistance was highest with *Acinetobacter*(9.65/), followed by *Citrobacter*(7.34%), *E.coli*(3.19%), *Klebsiella*(2%). The rate of emergence of resistance was highest for Amikacin[13.87%], followed by Nalidixic acid[6.62%], Cephalosporins[5.58%], Norfloxacin[4.5%], Piperacillin-Tazobactum[4.5%], Cefoperazonesulbactum[4.25%], Ampicillin[4.06%], and Nitrofurantoin[2.25%].

**Conclusion:**

Prevalence and resistance pattern changes every day. Practitioners should get acknowledged about the current trend of prevalence, sensitivity and resistance. Steps should be taken at all levels to prevent the emergence and spread of resistance.

**563**

**A_3_ and A_2a_ adenosine receptors as targets in cancer therapeutics**

Tote S^1^, Ghosh R^1^, Kulkarni JS^1^, Kadam VJ^1^

**^1^Bharati Vidyapeeth’s College of Pharmacy, Sector-8, C. B. D. Belapur, Navi Mumbai, India.**

Cancer is becoming a major health problem for entire world population and the currently available drugs for its treatment have clear limitations. Adenosine receptors have been the focus of many investigations as the sites for therapeutic intervention, including cancer. Extracellular adenosine exerts its effect through four subtypes of adenosine receptors. Stimulation of A_3_ adenosine receptor has been demonstrated to influence cell death and proliferation. Both adenosine and the A_3_ adenosine receptor agonists inhibit the growth of various cancer cell types such as melanoma, colon or prostate carcinoma, breast cancer and lymphoma. Preclinical and clinical studies on A_3_ receptor agonists viz IB-MECA, Cl-IB-MECA, LJ-529, LJ-568, MRS3558, MRS1898, CF101, cordycepin, ABEA-X-BY630, I-ABA have demonstrated anticancer activity and will be discussed in detail. The molecular pathways involved in the A_3_ receptor mediated anticancer activity will also be discussed. Besides A_3_ receptors, A2A receptors have also been postulated in mediating anticancer activity and will be discussed here in brief. Thus, both agonists of A_3_ and A_2A_ adenosine receptors are a promising new class of agents in cancer therapy.

**564**

**Cost analysis of medical therapy of glaucoma in India: A pilot study in pharmacoeconomic analysis**

Garcha SC, Gupta AK, Singh G, Garcha GS, Goyal D

**Government Medical College, Patiala, India.**

**Aim:**

To determine and compare the calculated annual costs of various topical anti-glaucoma medications.

**Study design:**

Experimental cost minimisation analysis.

**Method:**

We measured the actual volume of various commercially available glaucoma medications and average number of drops contained in each vial. Using their maximum retail price, we calculated theoretical cost per year of each drug for commonly recommended dosage. All medicines were assumed to be equally efficacious for comparison.

**Results:**

Among single salt agents, Optipress (Betaxolol) is the most economical anti-glaucoma medication (Rs. 273.7 ± 4.65 per year) while Xalatan (Latanoprost) is the most costly one (Rs. 8502.69 ± 281.77 per year). As a group, beta-blockers are most economical, costing between Rs. 273.7 ± 4.65 for Optipress to Rs. 704.96 ± 10.87 for Betagan (Levobunolol) per year, while prostaglandin analogues are most costly, costing between Rs. 2534.99 ± 29.02 for Lumigan (Bimatoprost) to Rs. 8502.69 ± 281.77 for Xalatan per year. Pilocarpine costs Rs. 1574.18 ± 38.31 per year. Alpha agonists cost between Rs. 1978.3 ± 99.43 for Alphagan (Brimonidine) to Rs. 2477.72 ± 79.52 for Alphagan P (Brimonidine) per year. Carbonic anhydrase inhibitors cost between Rs. 3215.89 ± 59.13 for Dorzox (Dorzolamide) to Rs. 4457.83 ± 143.51 for Ocudor (Dorzolamide). Yearly cost of various combinations vary from Rs. 1277.8 ± 53.67(Combigan - Brimonidine + Timolol) to Rs. 10341.34 ± 166.88(Xalacom - latanoprost + timolol).

**Conclusion:**

Beta-Blockers remain the most inexpensive group of glaucoma drugs. Cost of medications used may influence the decision making process clinically.

**565**

**Functional implication of p-glycoprotein (P-gp) modulation on the intraocular disposition of its substrate using intravitreal microdialysis**

Velpandian T, Senthilkumari S, Biswas NR, Saxena R, Ghose S

**All India Institute of Medical Sciences, New Delhi, India.**

**Purpose:**

To evaluate the functional role of P-gp and ocular tissue distribution of intravitreally injected Rhodamine-123 (Rho-123) in the presence of P-gp specific blocker (GF 120918) in normal as well as rifampicin-fed rabbits using microdialysis and direct sampling technique.

**Methods:**

Intravitreal pharmacokinetics of Rho-123 was conducted in male New Zealand albino rabbits. Direct sampling and microdialysis were employed to study the disposition of Rho-123 in normal as well as rifampicin-fed conditions. Control animals received Rho-123 at the concentration of 350 ng in PBS (0.05 ml) intravitreally, and the blocker-treated group received GF 120918 intravenously at the dose of 3.5 mg/kg 30 min prior to intravitreal injection of Rho-123. In case of direct sampling, four eyes were enucleated at different time points, and ocular tissues and humors were stored at -86 degrees C until analysis by HPLC with fluorescence detection.

**Results:**

In direct sampling, the blocker group showed significant increase (2.6 fold) in the mean vitreous concentration of Rho-123. Other tissues like ret-choroid, iris, and cornea also showed significant increase in their mean concentration. Microdialysis did not significantly predict the changes observed with direct sampling. Rifampicin-fed rabbits showed a vitreous pharmacokinetic profile comparable with non-fed (control) animals, and the pharmacokinetic parameters were unaffected by the blocker pretreatment.

**Conclusion:**

Intravenously injected blocker significantly altered the ocular disposition of intravitreally injected P-gp substrate. Rifampicin pretreatment did not upregulate P-gp transporters of the retina to the extent to affect the intravitreal kinetics of Rho-123 significantly. We acknowledge AIIMS for providing financial support.

**566**

**Antipyretic activity of *Spilanthes acmella* in experimental animal models**

Chakraborty A^2^, Devi BRK^2^, RITA S^2^, Singh ITh^1^

**^1^Agartala Govt. Medical College, Agartala, Tripura, India, ^2^Regional Institute of Medical Sciences, Imphal, Manipur, India.**

**Objective:**

***Spilanthes acmella*** is an indigenous herb from the family Asteraceae. It grows throughout the tropics. The whole plant has been described to possess medicinal properties. The flowers are used to relieve toothache. The leaves and flowers have been found to possess analgesic and anti-inflammatory activities of Spilanthes acmella. The present study was undertaken to evaluate the antipyretic activity (APA) of *Spilanthes acmella* in experimental animal models.

**Methods:**

Aqueous extract of *Spilanthes acmella* (ASA) was tested for APA action in albino rats by yeast induced method described by Burn JH et al., 1952, with slight modifications at doses of 100, 200, and 400 mg respectively. Normal saline and Aspirin (300mg/kg) were used as control and standard drugs respectively. After measuring the basal rectal temperature, animals were given subcutaneous injection of 20% aqueous suspension of dried yeast in 2% gum acacia at a dose of 20ml/kg below the nape of the neck to induce pyrexia.

**Result:**

The mean initial basal rectal temperature in this study was 99.4 ± 0.42 °F to 99.9 ± 0.24 °F. The rise in temperature after 19 hours of induction was 101.0 ± 0.37 °F to 101.3 ± 0.32 °F. The test drug in the various doses reduced the rectal temperature significantly from the 1^st^ hour to the 3^rd^ hour respectively. However, the reduction in temperature at the 4^th^ hour was not found to be significant.

**Conclusion:**

The present study indicates that APA has significant antipyretic activity.

**567**

**A low-molecular weight protein NK-31 identified from Indian monocellate cobra (*Naja kaouthia*) venom has apoptotic effect on C6 (glioma) cells**

Debnath A^1^, Vedasiromoni JR^2^, Gomes A^2^

**^1^School of Tropical Medicine, Kolkata, India, ^2^Indian Institute of Chemical Biology, Kolkata, India.**

**Introduction:**

***Naja kaouthia*** or commonly Indian monocellate cobra, is one of the most venomous snakes found in northeastern India. NK-31 is a bioactive molecule identified from the crude *Naja kaouthia* venom, which has very potent anti-neoplastic, apoptotic, cytostatic effect on human leukemic cells (according to our other studies). The present study screens its effect on glioma cell-line (C6) *in vitro*.

**Methods:**

IEC, SDS-PAGE and HPLC, Amino acid sequencing, cell culture (*in vitro*), MTT assay, Fluorescent microscopy using florescent dyes, TUNEL assay. Statistical analysis using students “t” test.

**Results:**

The protein NK-31 from *Naja kaouthia* crude venom was purified to homogeneity by heat treatment of crude venom, followed by ion-exchange chromatography, SDS-PAGE and HPLC. Molecular weight of NK-31 was found to be 6757D and N-terminal 20 amino acid sequence was LKCNKLVPLFYKTCPAGKNL… IC50 of NK-31 was found to be 20 micro g/ml of C6 cells. MTT assay showed significant cytotoxic effect of NK-31 treatment (24hrs) on C6 cells. Fluorescent photographs showed induction of apoptosis in C6 cells after NK-31 (1/2 IC50) treatment. TUNEL assay of NK-31 treated C6 cells showed TUNEL positive cells.

**Conclusion:**

NK-31, a low M.W. protein has been identified from the venom of the Indian monocellate cobra. NK-31 (in micro g concentration) showed its cytotoxic effect on C6 cells and showed strong apoptotic effect. NK-31 is a compound identified from cobra venom of Indian habitat, and its study on brain cancer model is first of its kind. Though this study is in its nascent stage, further elaboration seems promising.

**568**

**Controlled release of neostigmine from electrospun poly (vinyl alcohol) nanofibers for interathecal injection in rat**

Yousefifard M^1^^2^, Ranaei-Siadat SO^3^, Hassanpour ezati M^2^

**^1^New Ideas Research Institute (NIRI), Tehran, Iran, ^2^Shahed University, Theran, Iran, ^3^Shahid Beheshti University, Tehran, Iran**

Neostigmine is an anticholinestrase inhibitor and direct nicotinic agonist and produces analgesic effects when given by intrathecal rout in human. However, one of the therapeutic uses of neostigmine is limited by short elimination half-life. Electrospun nanofibers are explored as drug delivery systems using neostigmine as a model drug. In this study neostigmine was incorporated into electrospun poly (vinyl alcohol) (PVA) nanofibers for assessment as a controlled delivery device. A significant decrease in nanofibers diameter was observed with loading neostigmine. The scanning electron microscopic images indicated that the neostigmine was well incorporated into hydrophilic PVA. Electrospun nanofibers were stabilized against disintegration in water by treatment with alcohol such as ethanol. The drug release behavior from the electrospun nanofibers and its inhibitory effects on acethylcholine esterase enzyme were also investigated. Neostigmine release from the electrospun nanofibers showed biphasic pattern, characterized by an initial burst release followed by a sustained release for PVA 5-8%(W/W).A sustained-release of neostigmine with a low initial burst over 1 week was achieved from PVA 6%(W/W) nanofibers. The analgesic effect obtained by activating cholinergic mechanisms, however, seems to depend on the experimental pain model utilized for its evaluation. The antinociceptive effect of interathecal neostigmine was examined in rats submitted concurrently to the tail flick and formalin tests.

**569**

**Hypoglycemic effect of holarrhena antidysenterica [HA] seed powder in streptozotocin induced diabetic rats**

Kumar S, Gari M, Sharma J

**Rajendra institute of medical science, Ranchi, India.**

**Introduction:**

The world is facing an explosive increase in the incidence of diabetes mellitus and cost effective complementary therapies are needed. The effects of seed powder of HA in normal and diabetic rat were studied. Material and method: Streptozotocin (50mg/kg body wt.) single dose was administered intraperitoneally to induce diabetes. Rat with moderate hyperglycemia (serum glucose > 200 to 300 mg/dl) were randomly divided into three diabetic groups (C,D,and E) of six each. Group A (normal control) received 5ml saline /kg. body wt. Group B normal rat received HA 350 mg/kg body wt. Group C (diabetic control) received 5ml saline/ kg body wt. Group D diabetic rat received HA 350 mg/kg body wt. Group E diabetic rat received glibenclamide 0.5 mg/kg body wt. All were given orally for four weeks. Blood sugar was estimated every week for four consecutive weeks both fasting and postprandial by (GOD/POD) method. Result: There was a significant reduction (p<0.01) in pre-prandial and postprandial glucose level in diabetic rat (group D) on day 7 onwards. Hypoglycemic activity of HA on normoglycemic rat was significantly reduced (p<0.01) particularly in postprandial state on 28th day. The glucose level in HA treated diabetic rat significantly reduced (p<0.01) from 7th day onwards and was (145 mg and 185 mg verses diabetic control 215 mg and 265 mg) respectively in fasting and fed state on 28th day Conclusion: Seed powder of HA has significant anti-diabetic activity.

**570**

**A simple setup for rapid screening of anti-tussive activity in mice**

Kumar G, Mehla J, Katyal J, Gupta YK

**Neuropharmacology Laboratory, Department of Pharmacology, All India Institute of Medical Sciences, New Delhi-29, India.**

**Background:**

Sulphur dioxide (SO2) induced cough is an animal model, which evaluate the cough suppressant activity irrespective of mechanism of cough. However, this method is not used widely because of the complicated setup required for production of sulphur dioxide in situ and providing a control/standardized exposure to experimental animals. We have standardized and developed a simple and reliable setup for determining anti-tussive activity using sulphur dioxide for inducing cough in mice.

**Materials and Methods:**

Mice of either sex weighing 40-50g were used. Sulphur dioxide was produced by chemical reaction between sodium hydrogen sulphite and sulphuric acid in a penicillin vial placed at the base of a dessicator. The mice were placed on a wire gauge and exposed to sulphur dioxide for 45 seconds. The mice were then removed and placed in a well-ventilated chamber for observation of bouts of cough. Cough bouts were scored independently by two different observers and mean bouts in five minutes determined. For standardization, codeine was taken as reference standard. They were divided into three groups. Each group consisted of six mice. Group 1 received no drug, group 2 was administered distilled water and group 3 was administered codeine (10mg/kg, p.o.). All drugs/ vehicle were administered in volume of 0.3 ml. 20 minutes before second exposure to sulphur dioxide. Each animal served as its own control and percent inhibition in number of cough bouts was recorded.

**Results:**

In normal controls, there was no significant change in number of cough bouts between the two exposures while mice with distilled water showed a reduction of 20.5%. Codeine (10mg/kg, oral) pretreatment group, cough bouts decreased by 73.5%.

**Conclusion:**

The method described above is simple and reliable and can be used for rapid screening of cough suppressant activity of different compounds.

**571**

**Open label, single period, parallel study to evaluate the correlation of parasitic infestation with eosinophilia and the impact of anti-parasitic treatment in human, adult, male subjects**

Manchanda H^1^, Tandon M^1^, Pillai KK^2^, Mullick P^2^

**^1^Ranbaxy Clinical Pharmacology Unit, New Delhi, India, ^2^F/o Pharmacy, Jamia Hamdard, New Delhi, India.**

Eosinophilia occurs in abnormal conditions and disease states. An open label, single-period, parallel, two-centric study was conducted to study the natural course of eosinophilia, estimate the frequency of eosinophilia, evaluate the correlation of parasitic infestation with eosinophilia and the impact of antiparasitic treatment in twenty-two human, adult, male subjects having absolute eosinophilic counts (AEC) >500/mm^3^ at Clinical Pharmacology Unit-Majeedia (Site I) and CPU- Nodia (Site II). Stool examinations were performed to detect the presence of parasites and were divided into eosinophilia with/without parasitic infection. Subjects without parasitic infection were followed for their natural course of eosinophilia. Subjects with parasitic infection were then treated with single dose of 400 mg albendazole (for helminthic infection)/2 g tinidazole (for protozoal infection)/combination (for helminthic and protozoal infection). Higher incidence of eosinophilia (16.98%) was observed at Site II as compared from Site I (12.76%). Cause of eosinophilia could be identified in 64% of the subjects (50%- parasitic infection, 14%- current smokers). 11 subjects were positive for either protozoal or helminthic infections except for one subject who had mixed infections. All the treated subjects became stool negative with the first treatment dose. Mean AEC and serum IgE decreased significantly (*P* value <0.05) upon treatment in the eosinophilic subjects having parasitic infection. All the subjects completed the study. Both eosinophilia and serum IgE elevation were reported as hallmarks of intestinal parasitic infections; AEC could be an early indicator for responsiveness to the treatment. Parasitic infections should be considered as an important cause of eosinophilia.

**572**

**The short-term and long-term clinical outcomes following coronary stenting**

Thakkar A^1^, Mehta A^1^, Patel B^2^, Raykundaliya D^2^

**^1^L.M. College of Pharmacy, Ahmedabad, India, ^2^Sardar Patel University, Vallabh Vidyanagar, India.**

**Objective:**

The objective of this study was to compare the longterm clinical outcomes of patients receiving DES vs BMS in a real world clinical practice. Materials and Methods: Patients from SAL Hospital for Cardiovascular Disease included from August 22, 2006 through 26 January, 2007 in a study. We performed an analysis of 346 patients enrolled in study comparing drugeluting stents (DES) with bare-metal stents (mean 1, 3, 6 and 12 month follow-up). Results: The mean patient age was 56.3 years; 83%were males; 42.7% had unstable angina and 40.8% previous myocardial infarction (MI). Risk factors included hypertension in 62.1%, hypercholesterolemia in 52.4%, current smoking in 32.0% and diabetis in 28.2%. The study population included 94 patients who received DES and 242 who received BMS. After adjustment, DES reduced target vessel revascularization (TVR) rates at 1, 3, 6, and 12 months compared with BMS (12-month rates: DES, 6.6%; BMS, 16.3%; difference, –9.7%; 95% confidence interval [CI], P <.001). The TVR benefit for DES increased among patients with multi-vessel CAD (1-vessel CAD: 80%; 2-vessel CAD: 17%; 3-vessel CAD: 3%). However, in the overall cohort there were no statistically significant differences in the composite of death or MI (P = 0.16). Conclusion: Patients receiving DES vs BMS in a clinical practice have lower TVR rates, albeit with less absolute benefit than those observed in clinical trials. There is a sustained reduction in the need for TVR after the use of DES. The risk of death is not showing significant difference.

**573**

**Prescription patterns of anti diabetic drugs in patients with type 2 diabetes mellitus in a tertiary care institute in andhra pradesh**

Hasamnis A^1^, Patil SS^1^, Jena SK^1^, Sinha S R^2^

**^1^GSL Medical College, Rajahmundry India.**

**Grant Medical College, Mumbai, India.**

This study was conducted with the objective to evaluate prescription pattern of antidiabetic drugs in patients with type 2 diabetes mellitus attending the Medicine outpatient clinic of GSL Medical General Hospital. A prospective study was conducted for two months (November and December 2007) by reviewing the prescriptions of patients with type 2 diabetes mellitus attending the Medicine outpatient clinic of GSL Medical General Hospital, Rajahmundry, Andhra Pradesh. A total of 1000 prescriptions were studied. The average number of drugs per prescription was 4.65 which included antidiabetic drugs and drugs for co morbid conditions. The prescribing frequency of oral hypoglycemic drugs was more as compared to that of Insulin preparations. The fixed dose combination of various oral hypoglycemic drugs was prescribed more as compared to Sulfonylureas and Biguanides and other oral hypoglycemic drugs prescribed alone and this difference was statistically significant (P < 0.05) Fixed dose combinations of the various oral hypoglycemic drugs are prescribed more and preferred over oral hypoglycemic drugs used alone in the management of type 2 diabetes mellitus.

**574**

**Drug utilisation study in patients of bipolar disorder at a tertiary care hospital in delhi**

Chatley P, Roy V, Gupta U, Jiloha R

**Maulana Azad medical college and associated hospitals, New Delhi, India.**

**Introduction:**

Despite the wide prevalence of bipolar disorder, (1-2%), there is a paucity of data of all the drugs used in actual practice in India. To evaluate the prescribing pattern of drugs used in patients suffering from bipolar disorder. Materials and Methods: A prospective study was conducted, in patients on maintenance therapy for bipolar disorder, attending the Psychiatry OPD of GB Pant Hospital New Delhi. Exit interviews were conducted using a predesigned, pretested, questionnaire. Prescriptions were analysed using prescribing indicators by World Health Organisation. Results: 100 patients (53% males, 47% females) ranging 18yrs to 60 yr in age were enrolled, varying in disease duration from 3months-26years. Lithium was the most commonly prescribed medication (88%). Monotherapy was seen in only 5% of patients, 95%of patients received concomitant drugs. These included antiepileptics (sodium valprote, carbamezipine, lamotrigene) 40%, antipsyschotics (olanzipine, resperidone, haloperidol, trifluperazine, chlorpromazine) 38%, antidepressants (amitryptaline, miansrine, setraline, citalopram) 24%, anti-anxiety lorazepam,clonazepam, nitazepam, zolpidem) 21%of the patients. 17.46% of patients were prescribed drugs by generic name and73%of patients were prescribed drugs from the hospital essential drug list. 2.2%of drugs were prescribed per encounter. 34%, 28%, 21% 10%, 1% of patients received two, three, four & five, six & seven drugs respectively. No inject-able drug was prescribed. Conclusion: Combination therapy for bipolar disorder is being prescribed to majority of patients. Although the newer drugs are being used, lithium still remains the mainline drug. Although most drugs are from hospital essential drug list, use of generic name is limited.

**575**

**Effect of nigella sativa oil on hepatotoxicity induced by antitubercular drugs in albino rats**

Jadhav RR, Ghadlinge MS, Chandane RD, Jaju JB, Md. Mateenuddin

**S.R.T.R. Medical College, Ambajogai, Dist Beed, India.**

**Introduction:**

Liver plays an important role in drug metabolism. Drug induced liver damage due to antitubercular drugs (ATD) is most common in India. Antitubercular drugs induced Hepatotoxicity is mediated through oxidative stress. The seed of Nigella sativa (NS) has been used traditionally for centuries and has both antioxidant and anti-eicosanoid effect. As per available studies there is no systematic work was done to evaluate hepatoprotective effect of Nigella sativa oil on Antitubercular drugs induced Hepatotoxicity. Hence present study was carried out. To evaluate the prophylactic and therapeutic effect of Nigella sativa oil on Hepatotoxicity induced by Antitubercular Drugs. Materials and Methods: Healthy albino rats of either sex weighing 150-250 gm were used Study was conducted in two parts. In each part Animals were divided in 3 groups (6 animals in each group) ATD- Isoniazid (27 mg/kg), Rifampicin (54 mg/kg) and Pyrazinamide (135 mg/kg) were given orally and NS oil 0.2 ml/kg IP were used. Part I – study for 30 days Group I- Control Gr II- ATD treated Gr III- ATD + NS oil Part II- Gr I- ATD for 30 days Gr II-ATD for 30days + No treatment from 31-50 day Gr III-ATD for 30days + NS oil from 31-50 day. At the end of 30 and 50 day biochemical tests and histopathological examination of liver was done. Result: Nigella sativa significantly prevent the fall of total Serum Protein. Nigella sativa significantly prevent rise in Serum alanine aminotransferase, Serum aspartate aminotransferase, Serum Alkaline phosphatase and Serum bilirubin which rise due to antitubercular drugs. Nigella sativa significantly reduced the score of degeneration, necrosis and fibrosis which rises due to antitubercular drugs. There was also evidence of regeneration. Conclusion: It was found that Nigella sativa oil significantly prevents as well as reverse Antitubercular drugs induced Hepatotoxicity. Thus Nigella sativa oil is having both prophylactic and therapeutic hepatoprotective potential.

**576**

**The intraocular pressure lowering efficacy and safety of latanoprost 0.005% once daily versus brimonidine 0.2% Twice daily in patients of primary open angle glaucoma or ocular hypertension**

Singh P, Grover A, Sidhu HK, Singh G, Gupta AK

**Government medical college, Patiala**

**Objective:**

To compare the intraocular pressure(IOP) lowering efficacy and safety of latanoprost (0.005%) once daily versus brimonidine (0.2%) twice daily ophthalmic solution in patients of primary open angle glaucoma (POAG) or ocular hypertension (OHT) Material and methods: In this prospective open randomised trial 60 patients of POAG/OHT having IOP >21 mm Hg fulfilling the laid down inclusion criteria were randomly allocated into two groups of 30 patients each. Group A received one drop of latanoprost 0.005% at 9pm once daily and group B received one drop of brimonidine 0.2% at 9am and 9pm for 12 weeks. Baseline IOP was measured by applanation tonometry on day 0 at 9am before administration of the study drug. On subsequent visits IOP was recorded at the end of week 4, 8 and 12. After 12 weeks mean pre-treatment IOP, mean post-treatment IOP and mean IOP reduction (percentage) were 24.7 ± 1.75 mm Hg, 17.03 ± 1.40 mm Hg and 7.67 ± 1.48 mm Hg(31.05%) for group A and 24.36 ± 1.95 mm Hg, 18.55 ± 2.21 mm Hg and 5.81 ± 0.97 mm Hg(23.85%) for group B respectively. Conclusion: IOP reduction with latanoprost was more than brimonidine on all visits and this difference was statistically significant. Thus latanoprost has better IOP lowering efficacy as compared to brimonidine with convenience of once daily dosing. Both the drugs were safe and well tolerated with minor side effects but these did not lead to discontinuation of the therapy in either group.

**577**

**Augmentation of hypoglycemic effect of insulin with silymarin in diabetic rats**

Srivastava RK, Sharma S, Savita V, Singal R

**Pt. B.D.Sharma, University of Health Sciences, Rohtak**

**Objective:**

To study the effect of silymarin a hepatoprotective drug on blood glucose levels. Methodology: Rats were divided in three main groups depending on blood glucose levels into group-1: having normal BGLs (80 – 110mg/dl), group-2: diabetic rats with hyperglycemia (BGLs > 350mg/dl) and group - 3: insulin treated diabetic rats having BGLs <103 mg/dl. Silymarin (50mg/kg) given orally to all rats of each group for 45 days. Blood glucose levels were estimated at the end of experiments. Results & interpretation: In group-1 silymarin treatment produced no changes in BGLs. In group- 2 hyperglycemic rats, silymarin treatment brought down BGLs significantly (p< 0.005). In group- 3 diabetic rats treated with insulin to normalize BGLs, silymarin if combined with insulin there was further fall in BGLs (65mg/dl) leading to development of hypoglycemia. Conclusion: Our study shows that silymarin has blood glucose level lowering property and enhances the efficacy of insulin requiring adjustment of insulin doses.

**578**

**Neurotoxic effect of ajinomoto antagonized by ketamine in albino mice**

Hussain M S

**Kamineni Institute of Medical Sciences, Hyderrabad, Andhra Pradesh, India.**

**Background:**

Ajinomoto, monosodium glutamate (MSG), a central excitatory neurotransmitter plays an important role in ischemic brain damage and neurodegenerative disorders; its role in depression is still inconclusive.

**Aims and Objectives:**

To study the role of MSG on motor coordination &depression in albino mice.

**Materials and Methods:**

Four groups of Albino mice (n = 6), weighing more than 25gms were selected and subjected to various tests for motor coordination (Rotarod test, catatonic response time and hang test) and depression (forced swimming test and tail suspension test).Group1,2,3 received monosodium glutamate in 0.5gm,1gm and 2gm/day orally. Group 4 received Ketamine 1mcg/gm body wt. I.P along with 2gms of MSG per day. All the tests were repeated after 1week of administration. The animals were weighed before & after the study.

**Results:**

The mean of average time in Rotarod test increased for group1 (23.6-40.4sec) and group2 (10.4-45sec) & catatonic response time decreased for group1(28.8-17.0sec) & group2 (16.6-11.25sec).Group3 showed decrease in Rotarod test(15.8-3sec)& increased catatonic response time (18.8-46.2).Hang test for neuromuscular strength remained unchanged. The immobility time for 5minutes in Tail suspension test increased for all groups, Group1 (176.2-180.8sec) group2 (160.2-273.8sec) group3 (95-300sec). The immobility time in forced swimming test for 5min increased, Group1 (50-75.2sec), Group2 (130-166sec), Group3 (97.5-186.2sec). Weight increased in all groups. The motor coordination has improved in group1&2 but shows severe deterioration in group3 which has modestly improved in group 4 after administering Ketamine. All the groups showed signs of depression. Results were analyzed by paired‘t’ test and were significant. Conclusion: MSG produced neurotoxicity & depression in albino mice.

**Conclusion:**

MSG produced neurotoxicity & depression in albino mice.

**579**

**[+]-Huperzine A acts by NMDA receptor antagonism and protects against diisoprpyl-fluorophosphate induced seizure/*status epilepticus* in rats**

Kurnick M, Coleman BR, Nambiar MP

**Walter Reed Army Institute of Research, Silver Spring, USA.**

The toxicity of organophosphorous (OP) nerve agents is attributed to their irreversible inhibition of acetylcholinesterase (AChE), which leads to excessive accumulation of acetylcholine (ACh) and is followed by the release of excitatory amino acids (EAA). EAAs sustain seizure activity and induce neuropathology due to over-stimulation of *N*-methyl-D-aspartate (NMDA) receptors. Using primary rat neuronal cell cultures, we demonstrated that natural (-)-Huperzine A (Hup A), a blood-brain barrier permeable selective reversible inhibitor of AChE, reduce EAA-induced cell death by interfering with glutamate receptor-gated ion channels. Although [-]-Hup A, the natural isomer, inhibits AChE approximately 1000-fold more potently than [+]-Hup A, both [-]- and [+]-Hup A block the NMDA channel activity similarly. Here, we demonstrate that the in vivo mechanism of action of [+]-Hup A involves NMDA antagonism. Rats implanted with radiotelemetry probes to record EEG, ECG, body temperature, and physical activity was treated with [+]-Hup A, im and 30 min later with 20 *µ*g/kg NMDA (icv). For post-exposure, rats were treated with [+]-Hup A 1 min after NMDA. Pre/post-exposure treatment with [+]-Hup A protects animals against NMDA-induced seizures. Also, the survival of NMDA-administered animals was increased following [+]-Hup A treatment. [+]-Hup A has no effect on EEG, heart-rate, temperature, physical activity, or behavior indicating a reduced risk of side effects, toxicity, or associated pathology. Our results suggest that [+]-Hup A blocks NMDA-induced excitotoxicity *in vivo* and protects against seizure/*status epilepticus (SE)*. We further demonstrate that in a diisopropylfluorophosphate model, pre/post-exposure treatment with [+]-Hup protect against seizure/SE.

**580**

**Short term efficacy and safety of dexamethasone as an adjunct to imipramine in depression**

Khosla PP^1^, Sambhi R^2^, Singh M^3^, Singh BS^3^, Sharma KC^3^

**^1^Gian Sagar Medical College and Hospital, Banur, Patiala, India, ^2^Caswell Clinic Glanrhyd Hospital Bridgend, Midglamorgan, UK, ^3^Govt. Medical College and Hospital, Patiala, India.**

**Objective:**

To assess short term efficacy and safety of oral dexamethasone 4mg per day for four days as an adjunct to imipramine.

**Materials and Methods:**

Adult patients fulfilling ICD- 10 criteria and Hamilton Depression Rating Scale Score (HDRS) of >20 were admitted. Exclusion criteria were pregnancy, history of psychotropic drugs or steroids, electroconvulsive therapy during the last one month and co-morbidity with psychotic features and active medical illness, endocrinopathy or contraindication to steroids. In this randomized, double blind, placebo controlled, add-on design the patients of severe depressive episode were divided in two groups. Group A patients were given imiparamine 75 mg daily during first week and 150mg per day during the second week in patients with HDRS score ≥15. All patients in group A were given add-on dexamethasone 4mg per day for first four days where as in group B a matching placebo was given along with imipramine treatment as given in group A. Informed written consent was obtained and project was approved by IRB. Patients were evaluated at base line and 3^rd^, 5^th^, 7^th^, 10^th^ and 14^th^ day. Mean % improvement in HDRS and ADI score was more in group A as compared to group B on day 5, day 10 and day 14. More patients in group A showed a positive response on day 10 and 14.

**Conclusions:**

Short term administration of dexamethasone 4mg per day for four days along with standard imipramine treatment brings in quick and greater relief in patients of depression.

**581**

**Nasal therapeutics to medical countermeasure against inhalation exposure to chemical warfare nerve agents**

Nambiar MP^1^, Sciuto AM^2^

**^1^Walter Reed Army Institute of Research, Silver Spring, USA, ^2^United States Army Medical Research Institute of Chemical Defense, Aberdeen Proving Ground, USA**

Inhalation exposure is a major route of chemical warfare agent (CWNA) exposure during wartime/ terrorist attack. Rapidly progressing respiratory failure through a combination of muscarinic, nicotinic and CNS effects is the primary cause of death following CWNA poisoning. Although respiratory disturbances play a central role in CWNA induced pulmonary toxicity, the nature of which is not well investigated and there are no nasal remedies. We have developed a microinstillation technology of inhalation exposure to assess respiratory toxicity and develop therapeutics against CWNAs. Microinstillation involves aerosolization of the CWNA in the trachea directly to the lungs similar to a vapor inhalation. Acute microinstillation inhalation exposure to sarin resulted in respiratory toxicity and lung injury with or without seizures. Blood oxygen saturation and pulse rate decreased rapidly depending on the concentration of sarin. Sarin exposure leads to trachea and lung edema and changes in multiple bronchoalveolar lavage fluid (BAL), cell number and cell death. A number of FDAapproved or in advanced development nasal therapeutics have been evaluated as post-exposure treatment against sarin induced respiratory toxicity and lung injury following exposure to lethal doses. Our data showed that blocking airway secretion is very crucial for survival. Direct application of nasal therapeutics without nasal secretion blocker is contraindicative. Combination of nasal secretion blockers and respiratory therapeutics further decrease the respiratory toxicity and reduce lung injury. Our long-term goal is to unravel the molecular mechanisms of respiratory toxicity and lung injury and develop effective therapeutics for post-exposure CWNA casuality management.

**582**

**Impact of technologies on the drug development process**

Goel N^1^, Chaturvedi S^1^, Goel A^2^

**^1^Maharaja Surajmal Institute of Pharmacy, Janakpuri, N. Delhi, India, ^2^Jubilant Organosys, Noida, U P, India.**

Recent trends in drug development unleash the application of genomics, molecular biology and pharmacology in achieving new targets for drug design in therapeutic areas. Fundamental changes in the drug discovery scenario and associated relentless financial pressures have opened up new opportunities and challenges for the drug industry that it has to still fully grasp. The new drug discovery and development process is a capital-intensive, cumbersome, and highly complex multidisciplinary activity, which requires a highly committed team of chemists, biologists, biochemists, pharmacologists, toxicologists, ADME scientists, analytical chemists, pharmacists and clinicians to design, discover and develop new drugs. The entire process of drug development involves numerous pitfalls and is subject to stringent medicine regulations. The development stage is the most exacting in terms of bringing a new drug to the patient’s bedside. So the technologies and approaches which reduce the time a drug spends in trials, reduce the number of patients in trials, or that make the data gathering and data analysis more efficient, can help in reducing the drug development cost and time. A number of technologies such as pharmacogenomics, bioinformatics, and Internet based technologies can and will significantly influence various phases of clinical trials of drug development, both in terms of better selection of patients and drugs for clinical trials but also in the more efficient collection and analysis of data from trials. Recently, Biomarkers and genetic tests are increasingly used to determine the clinical effectiveness and safety of a drug. New and existing technologies are likely to reshape the drug discovery and development process in the future. Their impact will change the way in which drugs are discovered and developed. The present study will elaborate on the newly introduced technologies and there overall impact on the drug development process.

**583**

**Effect of polyherbalformulation USHBA in experimentally induced nephrolithiasis in rats**

Venkateswarlu M, Balaraman R

**M.S.University of Baroda, Vadodara, Gujarat, India.**

**Introduction:**

There is a still a large scope for herbal drugs in the treatment of nephrolithiasis as the modern drugs induce side effects on long term use. A lot of work has been carried out by researchers on various plants to evident their effectiveness in nephrolithiasis. But still lots many are left which are used in the indigenous system but no systemic studies regarding their pharmacology have been carried out. One such polyherbal formulation USHBA claimed to be useful in the treatment of renal calculi. USHBA contains a mixture of different aqueous extracts of Hemidesmus indicus, *Zingiber officinale, Glycyrrhiza glabra, Terminalia chebula, Nelumbo muifer, Myristica fragrans and Citrus aurantifolia.* The present study was aimed to evaluate the anti-nephrolithiatic activity of USHBA.

**Materials and Methods:**

Male Sprague dawley rats (200-225 gm, n=6-8) in 3 group i.e. control, Ethylene glycol(EG) + vitamin D3 control, and USHBA control. Nephrolithiasis was induced by 0.75% EG and vitamin D3 (5 micrograms/ kg in 5% CMC solution). Oral administration of USHBA 500 mg/kg, 1000mg/kg every day was carried out for 30 days. Animals were sacrificed by cervical dislocation, blood was collected for serum biochemical estimations. Kidneys were excised out for histo pathological studies and to prepare tissue homogenate.

**Results and Conclusion:**

Administration of ethylene glycol+ vitamin D3 led to significant decrease in the super oxide dismutase, catalase, glutathione and significant increase in lipid peroxidation in kidney homogenate. Administration of ethylene glycol+vitamin D3 showed significant increase in calcium oxalate in kidney homogenate. There was a significant increase in creatinine and urea levels in serum, and also significant increase in kidney weight when compared to normal control. Treatment with USHBA significantly reduced elevated levels of calcium and oxalate count in Nephrolithiatic rats. Poly herbal formulation treatment reduced elevated levels of biomarkers of oxidative stress further., treatment with USHBA significantly decreased serum creatinine and urea levels. Histopathology of kidney confirms a reduction in CaO crystal deposition in the kidney. Hence, Polyherbal formulation USHBA showed significant anti-nephrolithiatic and anti-oxidant activity.

**584**

**Evaluation of antinociceptive activity of emblica officinalis in rodent models of nociception**

Bhatia J, Golechha M, Gupta Y K, Arya D S

**All India Institute of Medical Sciences, New Delhi, India.**

In the present study, the antinociceptive effect of hydroalcoholic extract of Emblica officinalis (HAEEO) was evaluated on spinal (tail flick) and supraspinal (hot plate) models of nociception in albino mice. An analgesiometer was used to perform the tailflick test. The tail-flick response was elicited by applying radiant heat to a point approximately 1/3 of length away from the tip of the tail and the time taken by the animal to remove the tail from the heat stimulus was recorded (cut-off time was 10 s). In the hot plate test, the mice were placed on the heated (55 + 10C) surface of the hot plate. The time between placement on the hot plate and the occurrence of licking of the hind paws, shaking or jumping of the surface to avoid thermal pain was recorded as the response latency (cut-off time was 45 s). The tail-flick response and also in the hot plate test the response was measured at different times, up to 90 min, after intraperitoneal administration of HAEEO (150, 300, 450, 600 and 1000 mg/Kg) or vehicle (normal saline). HAEEO produced a significant increase in the withdrawal response latencies in a dose-dependent manner in both the models of nociception. The maximal antinociceptive effect was observed at 30 min in all the doses tested in both the models. Also, the maximal antinocicepive effect was observed at the dsoe of 600mg/Kg. There was no further increase in response at the dose of 1000 mg/Kg. Thus, HAEEO has good central antinociceptive activity.

**585**

**Efficacy and tolerability evaluation of oxymetazoline and dexpanthenol combination in comparison with xylometazoline: next study**

Pophale RR, Jagde MV, Langade DG

**Grant medical College, Mumbai, India.**

**Introduction:**

Combination of nasal decongestants with Dexpanthenol, a precursor to pantothenic acid, is shown to have synergistic effect in allergic rhinitis. Additional epithelial protective property of Dexpanthenol may help in wound healing of post nasal surgery patients.

**Methods:**

An investigator-blind, randomized, controlled, phase IV clinical trial conducted in 100 patients with acute allergic rhinitis or post-nasal surgery patients. Patients received either Oxymetazoline 0.05% with Dexpanthanol 5% (OD) or Xylometazoline 0.1% (XO) nasal drops.

**Results:**

Relief from nasal congestion was significantly better in the OD group then in the XO group (mean nasal scores 1.24 vs 1.86). Significantly more improvement in sneezing and decrease in nasal discharge was seen in the OD group than the XO group. Nasal irritation in the OD group was significantly less as compared to XO group (0.38 v/s 1.12 on second day and 0.10 vs 0.36 on the fourth day). The recovery time for OD group was 1.08 hours, which was significantly (46 min) lesser than that of the XO group. Rebound congestion was significantly less in OD as compared to XO group (6.25% vs 82.98%). 93.75% of the physicians in the OD group and 51.28% in XO group reported response to therapy as good to excellent. 95.83% patients in the OD group and only 52.91% patients in the XO group rated tolerability to therapy as good to excellent.

**Conclusion:**

Oxymetazoline and dexpanthenol combination has a better efficacy, shorter recovery time, causes lesser rebound congestion and has better tolerability than xylometazoline.

**586**

**Gastroprotective effect of tectona grandis: Possible involvement of H+ K+ atpase inhibition**

Singh N, Maurya R, and Palit G

**Central Drug Research Institute, Lucknow, India.**

Tectona grandis (TG), an Indian Teak plant is known to possess various therapeutic properties. We evaluated the anti-ulcer activity of TG leaf extract against various acute [cold restraint (CRU), alcohol (AL), aspirin (AS) and pyloric ligation (PL)] gastric ulcer models in rats and duodenal (histamine HA) ulcer model in guinea pigs and also its ulcer healing effect against Chronic acetic acid induced gastric ulcer model. TG at a dose of (250mg/kg, p.o) showed potential anti-ulcer activity against CRU (66.77%), AL (78.11%), AS (66.63%), PL (61.02%), HA (66.65%) induced ulcer models respectively, and are comparable to standard drugs. TG accelerated ulcer healing by reducing the size of ulcer area after 10 day treatment. On bioassay-guided fractionation of TG leaf, only aqueous (AF) and butanolic (BuF) fractions inhibited the gastric lesions induced by CRU and AL model showing protection of 83.3%, 66.7% and 81.65%, 83.19% respectively, whereas hexane (HF) and chloroform (CF) fractions were ineffective. Further, its effect on H+K+ATPase activity in vitro was also determined. TG and its AF and BuF fractions significantly inhibited H+K+ ATPase activity with an IC50 of 499.3 *µ*g/ml, 92.75 *µ*g/ml and 69.03 *µ*g/ml. These results suggest that TG was found to possess anti-ulcerogenic activity which might be due to its antisecretory activity through inhibition of gastric H+K+ATPase and also possess ulcer healing properties. Collectively these findings provide pharmacological information on the therapeutic efficacy of TG leaf against experimental gastric and duodenal ulcer.

**587**

**Adverse drug reaction reporting of antipsychotic drugs in gauhati medical college and hospital, guwahati**

Shivashankar P, Lahkar M, Chandan NG, Gohain S

**Gauhati Medical college, Guwahati, India.**

**Objectives:**

To report and evaluate adverse drug reactions of Antipsychotic drugs.

**Methods:**

A cross sectional study was conducted involving 102 patients on Antipsychotics, in the Dept. of psychiatry, Gauhati Medical College & Hospital for a period of one month.

**Results:**

Of these 102 patients, 68 patients were treated with Olanzapine, 34 patients with Haloperidol. The adverse effects seen in patients on Olanzapine in the decreasing order of frequency are: increase in appetite (40nos), autonomic(29nos), weight gain(25nos), gastrointestinal symptoms(20nos), CNS(7nos), extra pyramidal symptoms(3nos). The adverse effects seen in patients on Haloperidol are: extra pyramidal symptoms(17nos), gastro intestinal symptoms(13nos), autonomic(10nos), CNS(7nos), endocrine(3nos), jaundice(1nos)

**Conclusion:**

The most common adverse effect seen with Olanzapine is, increase in appetite followed by autonomic effect and weight gain and extra pyramidal symptoms was found to be less common whereas in case of Haloperidol, extra pyramidal symptom was found to be the most common adverse effect.

**588**

**Preliminary results on carbon isotope ratios of endogenous steroids for establishing reference ranges in indian population**

Shweta S, Kaur T, Jain S, And Beotra A

**National Dope Testing Laboratopry, J.L.N.Stadium New Delhi, India.**

**Introduction:**

Use of anabolic androgenic steroids (exogenous & endogenous) by athletes has become widespread. Gas chromatography/Combustion/Isotope Ratio Mass Spectrometry (GC-IRMS) is a technique adopted for detecting and confirming the abuse of anabolic steroids (exogenous) which occur naturally in body (endogenous). Endogenous steroid profile is affected by interindividual and ethnic generic differences. The limited knowledge on the effect of androgenic doping on testosterone excretion in Asian population increases the risk of false negative results. Hence, there is a need to establish a reference range based on Carbon isotope ratios (δ13C values) of Indian population.

**Materials and Methods:**

Drug free urine samples (DFU) were collected from 100 Indian healthy male and female (aged 16-30 years). All the samples were processed using solid phase extraction, derivatised using acetylation and were subjected to GC-IRMS analysis.

**Results and Discussions:**

AS per WADA Technical document the results reported as consistent with administration of a steroid when δ13C values measured for metabolites differ significantly i.e. by 4 delta units or more from that of urinary reference steroid chosen. The δ13C values of endogenous androsterone and etiocholanolone (metabolites of testosterone) & 11-keto-etiocholanolone (used as Endogenous Reference Compound) ranged from -21.79 to -26.0 (±0.93), -22.57 to -26.75 (±1.13) & -17.83 to -26.89 (± 1.80).

**Conclusion:**

Comparison of δ13C values of a control group (reference range of DFU) with the data obtained from athletes established to be abusing endogenous steroids, will prove very useful in reporting any adverse analytical finding. This study will be extended to more number of samples of different ethnic origin to serve as a data bank for reference ranges for Indian population.

**589**

**Knowledge, attitude and perception of allopathic doctors about complementary and alternative medicine (cam) in a tertiary care hospital in india- an observational study**

Ghosh R, Roy V, Chatley P, Kaushik N

**Maulana Azad Medical College and Lok Nayak Hospital, New Delhi, India.**

**Introduction:**

(CAM) is a group of diverse medical and health care systems, practices and products that are not presently considered to be part of conventional medicine. CAM is being increasingly practiced all over the world. The objective of the study was to determine the knowledge, attitude, perception and extent of use of CAM amongst allopathic doctors in a tertiary care hospital.

**Methods:**

200 doctors of Maulana Azad Medical College & Associated Hospitals were interviewed with help of a pretested questionnaire which had questions pertaining to their demographic, socioeconomic status and also questions relating to their knowledge attitude and perception towards CAM.

**Results:**

Of the 200 doctors included in the study majority were male(58%), aged 20-30 years (54%), postgraduate resident doctors (70%). 58% doctors received CAM at least once and 50% actually felt better with it. Homeopathy, Ayurveda, Yoga were the most commonly used CAM for conditions like dermatitis, warts, baldness, bronchitis, back pain and arthritis. 52% doctors believed in the beneficial role of CAM and 40% recommended its use in patients. Majority doctors believed in the cost effectiveness (65%) need for awareness (67%) and less adverse drug reactions about CAM. Most of them feel unless trained, an allopathic doctor should not prescribe CAM and 62% opined MBBS students should be sensitized about CAM. 31% of reviewed doctors believed drug interactions between allopathic and herbal medicines can occur and 34% were aware of different ongoing clinical trials on CAM.

**Conclusion:**

CAM is commonly practiced amongst allopathic doctors. Knowledge and awareness about CAM and Integrated medicine need to be spread for more holistic healthcare in our country.

**590**

**Monitoring of adverse drug reactions to antiretroviral drug therapy in aids patients in a tertiary care hospital – A prospective observational study**

Nagpal M, Gupta U, Kumar S

**Maulana Azad Medical College and Lok Nayak Hospital, New Delhi, India.**

**Introduction:**

Highly Active Antiretroviral Therapy (HAART) is the corner stone of management of patients with HIV infection. Antiretroviral drugs are associated with various adverse drug reactions (ADR). The objective of the study was to monitor the different types of ADRs in patients receiving antiretroviral therapy.

**Methods:**

Adult patients receiving antiretroviral therapy were followed at monthly intervals for a period of 6 months. The ADR monitoring was done in a systematic manner adopting spontaneous and intensive monitoring approach. The type of ADR, its severity and impact on compliance was recorded. Causality assessment and severity grading of ADR was done using WHO guideline.

**Results:**

A total of 235 patients were included in the study. 162 patients (68.94%) received Treatment Regimen A (Stavudine, Lamivudine, Nevirapine), 41patients (17.45%) received Treatment Regimen B (Stavudine, Lamivudine, Efavirenz), 26 patients (11.06%) received Treatment Regimen C (Zidovudine, Lamivudine, Nevirapine)and 6 patients (2.55%) received Treatment Regimen D (Zidovudine, Lamivudine, Efavirenz). 213 patients (90.64%) patients reported ADRs. A total of 618 ADRs were observed. 403, 143, 43 and 15 ADRs were observed in treatment regimens A, B, C and D respectively. 41 ADRs (6.64%) were assigned probable and 577 ADRs (93.36%) assigned to possible causality assessment. 188 ADR (30.42%) were mild in severity, 287 (46.44%) were moderate and 143 (23.14%) were severe. 68 patients (28.94%) were noncompliant to treatment due to ADRs.

**Conclusions:**

Antiretroviral drugs are associated with many adverse drug reactions which often lead to non-compliance. Therefore, antiretroviral drugs should be used judiciously and patients should be monitored for adverse drug reactions.

**591**

**Analgesic and anti-inflammatory activity of ethanolic extract of capparis decidua fruit extract**

Goyal M.^1^, Nagori B.P.^1^, Sasmal D^2^

**^1^Lachoo Memorial College Science and Technology Pharmacy Wing, Jodhpur, Rajasthan, India, ^2^Department of Pharmaceutical Sciences, BIT mesra, Ranchi, Jharkhand, India.**

Capparis decidua (frock) Edgew (family: Capparidaceae.), commonly known as karrel or ker, have been reported to have the antioxidant and hypolipidemic activity. In a previous study, the aerial part of plant extract prevented carrageenan induced edema, in present study a battery of tests such as acetic acid induce writhing, tail flick, and carrageenan induced inflammation were carried out using ethanolic extract of fruit of Capparis deciduas to assess analgesic and anti-inflammatory activity of fruit part. Significant (P < 0.001) decrease in paw edema induced by carrageenan were observed at all dose level i.e. 100 mg/kg, 200 mg/kg and 300 mg/kg when compared with control, highest activity was showed at 300 mg/kg p.o. inhibited inflammation by 69.89% which is not significant when compared with 100 mg/kg p.o. phenylbutazone which inhibited inflammation by 80.64%. In the tail-flick method, significant (P < 0.05) elevation in mean basal reaction time was observed when compared with control. Antinociceptive activity (%) was 33.33, 46.96 and 68.18 for respective doses of 100, 200 and 300 mg/kg. In acetic acid induced writhing model, ethanolic extract inhibited no. of writhes produced, which were calculated as percentage protection, showing maximum protection at the dose of 300 mg/kg. The extract showed prevention against the writhing induced by acetic acid as well as against carrageenan induced inflammation and tail-flick. The results from present study provide further evidence about the involvement of higher centers in the analgesic activity of the plant extract. Further studies are warranted to elucidate the mechanism of analgesic effect.

**592**

**Herbal toxicity in children: An overview**

Awatade N T, Gorule A A, Vyawahare N S

**AISSMS College of Pharmacy, Kennedy road Pune-411001, India.**

Many herbal remedies are self-administered by adults without any guidance from knowledgeable sources as to their indications, efficacy, or safety. Parents may be tempted to give combinations of herbs to children on the basis of advertising for the products, information that they may glean from a magazine or web site, or advice from friends or relatives. Such experimentation is expensive and risks exposure of the child to unwanted adverse effects. Adverse events associated with herbal medicine typically results from long term use at inappropriate dosage levels, the use of certain highly toxic substances and hypersensitivity reactions. Also herbal medicine probably presents a greater risk of adverse effects and interactions than any other therapy. Children differ from adults in their absorption, distribution, metabolism, and excretion of some substances. They also have developing central nervous and immune systems that may make them more sensitive to the adverse effects of herbs. Infants and young children are physiologically more vulnerable to certain adverse effects of herbs than are adults. In this review we have discussed some herbal drug toxicities such as Garlic, Ginseng, Gingko, Hawthorn, Kava, Licorice, Uzara root, Hawthorn etc. and examples of potential drug-herb interactions and there is an urgent need to study in detail such toxicities.

**593**

**Antioxidants mediate radioprotection: Is combination better than solo?**

Alok A, Mishra K N, Ojha H, Anuradha, Athar Md, Simhachalam K, Lahiri S S, Bhatnagar A, Manda K, Chaudhury N K, Adhikari J S

**Institute of Nuclear Medicine and Allied Sciences, Lucknow Road, Timarpur, Delhi -110054. India.**

The knowledge of antioxidants as radio protector in vitro and in vivo is well established. Antioxidants primarily act by scavenging free radicals produced by ionizing radiation. The use of antioxidant as a potential radio protector in case of untoward nuclear accidents is constrained by their requirement in high doses. For example, melatonin and lipoic acid is required in the range 200-250 mg/ kg of body weight in order to be radioprotective in mice. We have observed promising result in eight week old Swiss albino mice which was administered melatonin in combination with other antioxidants like alpha lipoic acid and ferulic acid. A whole body lethal radiation dose of 8.5 Gy was delivered using a 60Co source at the dose rate 0.54 Gy/min. We are investigating the reason for high dose requirement of melatonin through spectroscopic methods in physiological solution (PBS), ethanol, DMSO and their mixture. The melatonin in solution precipitates after 12 hrs at controlled humidity, pressure and temperature. The absorbance and fluorescence characteristics decreases in the solvent portion after 12 hrs. which appears to be one of the contributory factor for the poor bioavailability of melatonin in the physiological system and therefore, combination of melatonin with other antioxidants has potential for better efficacy in comparison to solo administration. The antioxidants show better radioprotection when injected with 5% DMSO in PBS which is because DMSO helps in sustained release of drug besides having intrinsic antioxidant property.

**594**

**Effect of ionization on membrane transport: A dilemma**

Muhammad Akram Randhawa, Talay Yar

**College of Medicine, King Faisal University, Dammam, Saudi Arabia**

Most drugs are weak bases or acids and are present in solution as unionized and ionized species. Unionized molecules are lipid soluble so easily diffuse across membranes, while ionized molecules are less lipid-soluble and difficult to cross. Ratio of unionized to ionized (U/I) forms of a drug at each pH can be calculated from Henderson-Hasselbalch Equation (HHEq). Thus, HHEq helps to anticipate transmembrane distribution of a weak electrolyte when its pKa and pH of medium is known. HHEq also said to help in the anticipation of influence of pH on renal excretion (RE) of drugs. At equilibrium total drug (unionized + ionized) will be different across the membrane: a basic drug will concentrate in compartment with low pH, and vice verse for an acidic drug (.‘ion trapping’). According to HHEq, drugs with similar pKa have equal ratios U/I at same pH of medium, thus equal capacities to cross membranes. However, drugs with similar pKa values at same pH had different capacities to diffuse across membranes: a) Ratio U/I of 10 basic drugs (with similar pKa, around 9) at pH 9 and % Buccal Absorption (BA) at pH 9 showed poor correlation (r=0.19 % P=0.58); b) ratio U/I of 10 acidic compounds (with similar pKa, around 4) at pH4 and % BA at pH 4 showed poor correlation (r=0.45 & P=0.19); c) ratios of 24-hour RE in acid and alkaline urine (ration Ac/Al) of 10 basic drugs with similar pKa values (around 9) varied from 1 to 99 and their pKa and ratios Ac/Al were not correlating (r=0.039 & P=0.915). In a recent study, two β-blockers (Atenolol & Metoprolol) with similar pKa (9.6 & 9.7) had widely different permeability coefficients (186 ± 6×10-6 cm s^-1^ and 2.31 ± 0.09 ×10^-6^ cm s^-1^, respectively) at similar pH of the medium, pH 8 apical side and pH 7.4 basolateral side, when passive diffusion was examined across Caco-2 cell monolayers. Quaternary amine muscarinic and anti-muscarinic drugs which are ionized in solution and poor to cross membranes were equivalent to tertiary amine muscarinic and anti-muscarinic drugs for their action on pupil size in rabbit eye; in terms of their onset and duration of action and relative potencies. We conclude that ionization, as calculated from HHEq, generally does not correlate with membrane transport of weak electrolytes. ‘Ion trapping’ does occur but varies greatly amongst individual basic or acidic drugs, despite similar pKa values and pH of the medium. Recently, other factors have been proposed to influence passive diffusion like opening of tight junctions in paracellular route.

**595**

**Pharmacovigilance in respiratory medicine: An experience with theophylline**

A Ray, Kavita Gulati, N Taygi, G Vishnoi, VK Vijayan

**Vallabhbhai Patel Chest Institute, University of Delhi, Delhi, India.**

**Objective:**

The present study evaluated the adverse drug reaction (ADR) profile of theophylline in obstructive airway disease, and attempted to analyze the possible mechanisms involved in its toxicity.

**Methods:**

Outpatients of bronchial asthma and COPD were screened at the VPCI according to inclusion and exclusion criteria. All patients prescribed standard oral theophylline as part of asthma and COPD therapy were assessed for ADRs and the data was recorded, compiled and analysed for causality using the Naranjo s scale. In the next part of the study, the toxicodynamics of a prominent theophylline–induced adverse effect was evaluated in experimental animals with an aim to predict strategies for effective prevention/management of such theophylline induced ADRs. The data was statistically analyzed by standard nonparametric tests.

**Results:**

In the clinical study, 60 patients each of bronchial asthma and COPD were observed, out of which 33% and 71% received theophylline, respectively. 46% of COPD patients showed ADRs with theophylline, as compared to 71% in asthma patients. The overall ADR profile was : anxiety (34%), dyspepsia (33%), muscle spasm (21%), insomnia (17%) and others (22%). Causality assessment showed that most ADRs fell in the “probable” category. Experimental studies in rats, in the elevated plus maze test, showed that theophylline induced anxiety could be significantly (*P*<0.05) attenuated by the antioxidants (alpha tocopherol and melatonin).

**Conclusions:**

These data suggest that theophylline induced neurobehavioral toxicity can assume significant proportions in obstructive airway disease, and that prophylactic anti-oxidant therapy could be beneficial in its prevention.

**596**

**Effects of propoxur on cognitive function and oxidative stress and its modulation by melatonin, progesterone and 4’- chlordiazepam in rats**

Khanna N, Mehta KD, Sharma KK, Tripathi AK

**University College of Medical Sciences, Department of Pharmacology, New Delhi, India.**

Effects of propoxur on cognitive function and oxidative stress and its modulation by melatonin, progesterone and 4’ - chlordiazepam were studied in rats. Oral administration of propoxur (10mg/kg) increased lipid peroxidation in brain after 6 weeks of treatment. Malondialdehyde, glutathione, catalase were also altered following propoxur exposure. In addition propoxur caused marked cognitive impairment as assessed by step down latency and elevated pus maze. After 6 week of propoxur administration melatonin (50mg/kg, ip), progesterone (10mg/kg, ip) and 4’ - chlordiazepam (0.5mg/kg, ip) were administered for 1 week. Melatonin and 4’ - chlordiazepam attenuated the effect of propoxur on cognitive function and oxidative stress parameters, where as progesterone failed to do the same. Results have been discussed in the light of possible antioxidant effects of Melatonin and 4’ - chlordiazepam to understand the influence of oxidative stress on propoxur induced cognitive function.

**597**

**Anti-diabetic and anti-dyslipidemic effect of 1CDED88D extract in a murine model**

Jain S, Khanna V, Roy S, Surendar A, Yadav S, Kanaujia A, Mittra S, Davis JA, Bansal VS, Katiyar CK, Ray A, Bhatnagar PK

**University College of Medical Sciences, Department of Pharmacology, New Delhi, India.**

**Introduction:**

Current antidiabetic agents have caveats and are associated with side effects. Herbal anti-diabetic extracts are promising as a unique approach for meeting the need for safe, effective and relatively inexpensive new remedies for prolonged use in treatment of type 2 diabetes and its related disorders. The present study describes an extract, ICDED88D, which has shown *in vitro* and *in vivo* efficacy against type 2 diabetes.

**Methods:**

In vitro anti-diabetic activity was evaluated by glucose uptake in differentiated L6 myotubes, and RT-PCR to characterize the mRNA expression of PI3 Kinase and Glut4. In vivo efficacy was established in ob/ob mice by oral administration of ICDED88D (500 mpk bid) for 21 days.

**Results:**

The extract showed insulinomimetic activity as evidenced by increased glucose uptake by L6 myotubes and a significant increase in PI3-kinase & GLUT4 mRNA expression. Further, oral administration of ICDED88D caused significant glucose lowering (27.7%), comparable to rosiglitazone (32.3%). The extract also exhibited significant decrease of 27.6% in AUC0- 120 min following oral glucose load. In addition, the extract showed a significant reduction in plasma triglyceride level (30.5%). Interestingly, ICDED88D extract did not alter the body weight of diabetic animals when compared to a significant increase of body weight by rosiglitazone.

**Conclusion:**

ICDED88D possesses significant anti-diabetic and hypo-triglyceridemic activity and has the potential to be used as a standardized extract for the treatment of Type 2 diabetes and related complications.

**598**

**A comparative study of efficacy of inhaled aminoglycoside tobaramycin in COPD patients**

Sharma KK, Sharma L

**SMS Medical College Jaipur**

**Objective:**

Efficacy of inhaled aminoglycoside tobramycin in COPD patients.

**Method:**

This study was conducted on patients suffering from COPD with frequent chest Infection since last 10 years. In this study indoor patients of 50-70 years were selected and divided into two groups of fifty of each this study was done in private hospital at Jaipur.

**Group 1:**

Patient was treated by with conventional Bronchodilator like beta agonists and anticholinergics. In addition to oxygen therapy and oral antibiotics.

**Groups 2:**

Patients were treated with nebulisation with tobramycin 300mg twice daily In addition to conventional Bronchodilator oxygen and similar oral antibiotics For symptomatic relief other drugs like paracetamol, proton pump inhibitor antiemetics were also prescribed. The progression of treatment was monitored by serial estimation of total leucocytes count, ESR chest x-ray, Quantity of sputum production daily, Pulmonary function test on alternate day, oxygen saturation by pulse oxymeter, Arterial blood gas analysis, clinical parameter like respiratory rate, blood pressure, fever, patient wellbeing.

**Result:**

This study showed that group -2 patients showed improved out come in terms of less duration of hospital stay (5 days in group-2 in compared 7 days in group-1) Decreased quantity of sputum production, early clearance of chest X-ray, Saturation >92%, Rapid normalization of cell counts,Patient wellbeing in comparison to group -1 patient. Pulmonary function test showed rapid improvement in functions like increase in FEV-1 in Group-2 patients showed less frequency of hospital admission.

**Conclusion:**

Inhaled aminoglycoside tobramycin therapy showed significant improvement in COPD patient in terms of less duration of hospital stay and improvement in pulmonary function tests.

**599**

**Comparative efficacy of nebivolol and atenolol on hypertensive status and oxidative stress in hypertensive patients**

Kalavathi D^1^, Uday V^2^, Venkat Rajaiah N^3^, Santrani^1^, Rama Krishna D^2^

**^1^Padmavathi Womens College,Thirupati, ^2^Kakatiya University, Warangal, ^3^Kakatiya Medical College,Warangal,India.**

Hypertension is a common disorder that, if not effectively treated, results in a greatly increased probability of coronary thrombosis, renal failure, and stroke. Atenolol is a hydrophilic β-1 selective adrenergic receptor blocker. Nebivolol is a newly developed beta-adrenergic receptor blocking drug. Healthy volunteers, male patients of age 30-60 years, with normal blood sugar levels and patients with only essential hypertension with out any prior antihypertensive therapy were included in the study. Each group comprised of 20 hypertensives (n=20).We demonstrated that the novel beta-1 receptor blocker nebivolol has potent inhibitory effects on vascular superoxide production. Nebivolol improved total antioxidant property, and improved Glutathione levels. The present study indicates that the selective beta-1 receptor blocker nebivolol is able to decrease MDA levels by scavenging superoxide ions and thereby increasing intracellular GSH levels. We conclude that nebivolol appears to be lipid neutral and may have a positive effect on blood sugar and HDL cholesterol.

**600**

Singh GD^1^, Singh S^1^, Youssouf MS^1^, Sharma R^1^, Pagoch SS^1^, Sharma SC^1^, Chandan BK^1^, Koul A^1^, Sangwan PL^2^, Gupta BD^2^, Koul SK^2^, Johri RK^1^

**^1^Division of pharmacology, Indian Institute of Integrative Medicine (CSIR) Jammu (J&K), India, ^2^Division of Natural products Chemistry, Indian Institute of Integrative Medicine (CSIR) Jammu (J&K), India.**

The purpose of this study were to evaluate the safety of aqueous extract of Labisia pumila. The aqueous extract of Labisia pumila (LP) was studied for its acute and subacute toxicity in Swiss mice and Wistar rats respectively. The LD50 value of LP was found to be more than 5000 mg/kg per-oral. Following the chronic administration of LP for 28 days in different doses of 12.5, 25 and 50 mg/kg, the body weight gain, organ/body weight ratio, food and water intake were recorded. The hematological and biochemical parameters at the end of the study were determined. The vital organs such as heart, liver, kidney, testis, ovary, spleen, brain, stomach were subjected to histopathological studies and any apparent & significant changes or differences from the normal were recorded. The results did not show any potential side effects. In the acute toxicity test, oral administration of 5 g/kg of Labasia pumila produced neither mortality nor changes in behavior or any other physiological activities. In subacute toxicity studies, no mortality was observed when the doses of 12.5, 25 and 50 mg/kg/day of LP were administered orally for a period of 28 days. No significant changes occurred in the blood chemistry analysis including glucose, creatinine, blood urea nitrogen (BUN), aspartate transaminase (AST), alanine transaminase (ALT), total billirrubin, total cholesterol, triglycerides and total protein of both sexes. Hematological analysis showed no differences in any of the parameters examined (WBC count, RBC count, platelet, hemoglobin, hematocrit, lymphocyte, monocyte, and granulocyte) in either the control or treated group of both sexes. The urinalysis was negative for glucose, albumin, bile salts, bile pigments, ketonic bodies, casts, red blood cells, leukocytes, epithelial cells and calcium oxalate crystals in the control and treatment groups. There were no significant differences in the body and organ weights between controls and treated animals of both sexes. Pathologically, neither gross abnormalities nor histopathological changes were observed.

**601**

**DRDE-07 and its analogues as promising cytoprotectant to nitrogen mustard (HN-2): An alkylating anticancer and chemical warfare agent**

Manoj Sharma, R Vijayaraghavan, S.C Pant, K Ganesan

**Defence Research and Development Establishment, Gwalior (M.P.), India.**

**Objective:**

Nitrogen mustard (HN-2) or Mechlorethamine is well known alkylating anticancer agent as well as blistering chemical warfare agent. The aim of the study was to evaluate cytoprotective efficacy of amifostine, DRDE-07 and its analogues, and other antidotes of mustard agents against HN-2 (mechlorethamine) at different sublethal doses.

**Materials and Methods:**

Single LD50 dose of HN-2 by percutaneous route was used to evaluate toxicity pattern of HN-2. Repeated dosing of antidotes at one equi. molar dose of DRDE-07 for 3 and 7 day were administered against percutaneously applied single dose of 0.5 LD50 and 0.25 LD50 dose of HN-2 and various cytoprotective parameter were evaluated.

**Results:**

The WBC count abruptly decreased form 24 hours onwards. GSH level decreased prominently and maximum decrease was observed on 7th day post administration. GSSG content increased significantly at 24 hrs post administration and subsequently decreased in progressive manner. The MDA level and percent DNA damage was also progressively increased. The spleen weight decreased progressively and reached a minimum on 3 to 4 days and then started increasing. DRDE-07, DRDE-30 and DRDE-35 partially and significantly protected the change in the same histological, biochemical and histopathological parameters following HN-2 administration at 0.5 LD50 and 0.25 LD50 dose level respectively.

**Conclusion:**

The present study shows that nitrogen mustard (HN-2) is highly toxic by percutaneous route and DRDE-07, DRDE-30 and DRDE-35 partially mitigated the toxicity of HN-2.

**602**

**Gene expression profile and biochemical alterations of antioxidant enzymes in mice following t-2 toxin exposure**

Chaudhary M, Jayaraj R, Santhosh SR, Rao PVL

**Defence Research and Development Establishment, Gwalior, India.**

T-2 is a cytotoxic fungal secondary metabolite, produced by various species of Fusarium, contaminating foods, animal feed and agricultural products. The study was conducted to examine biochemical and gene expression changes induced by T-2 toxin on antioxidant enzymes of mice treated intraperitonially with 1LD_50_ (5.61mg/kg) and 2 LD_50_ (11.2mg/kg) doses. T-2 toxin induced oxidative stress in mice, as seen by alterations in levels of antioxidant enzymes and expression of HSP-70. Changes were determined after 4h and at time to death. A significant time dependent increase in HSP-70 expression over control was observed at 2 LD_50_ dose. There was a dose dependent increase in hepatic liver peroxidation at both the doses. Depletion in GSH was seen at 2 LD_50_. Except for GR, there was a significant increase in activity of other antioxidant enzymes GST, GPx, SOD and catalase at 1 LD_50_. At 2 LD_50_ GR and SOD showed depletion while GST, GPx and catalase showed a significant increase. In contrast to activity of antioxidant enzymes, real time gene expression profile showed a down regulation in GPx at 2 LD_50_ while others i.e GST, GS and GR showed an up-regulation. Similarly for 1 LD_50_, GS and GR showed up regulation whereas GPx and GST showed an initial elevation, again depleting significantly at the time of death. The results of this study show that oxidative stress is induced by T-2 toxin and also that biochemical changes were not always in accordance with changes in expression at gene level.

**603**

**Selection of anesthetic agent to achieve prolong stable blood pressure in rats**

Gupta R^1^, Mathur A^1^, Mohanan A^1^, Joshi D^>1^

**^1^Torrent Pharmaceuticals Ltd., Research Centre, Gandhinagar, India.**

Effect of long term anesthesia on laboratory rodents can be a confounding factor in experimental findings involving anesthetized animal model. This is specially applicable for anesthesia lasting for period of more than 1hr. We compared suitability of intravenous ketamine-xylazine infusion or isoflurane inhalation as anesthetic agent. Stable blood pressure (BP) over the period of anesthesia was used as an indicator of physiological stabilization. Sprague Dawley rats were anesthetized either with intraperitonial injection of ketamine-xylazine combination (60+7.5mg/kg, respectively) or in induction chamber with 6% isoflurane diluted in medical oxygen. While anesthetized, body temperature was maintained at 37°C. Rats were tracheostomized and were artificially ventilated either with room air or with 1.5% isoflurane in medical oxygen. The jugular vein and femoral artery were cannulated for drug administration and measurement of arterial BP, respectively. After completion of the surgical procedure, mecamylamine and atropine were administered to inhibit autonomic nervous reflexes and then isoflurane inhalation was readjusted to either 0.8% or 1% or ketamine-xylazine cocktail infusion at dose of 1250+40*μ*g/kg/min was initiated after 45min of initial induction dose of ketamine-xylazine combination. While under maintenance anesthesia with either anesthetic, BP was recorded continuously for 6hr. Ketamine-xylazine combination though found successful in maintaining continuous surgical plain of anesthesia caused gradual fall in BP beyond 3hr of continuous infusion. Isoflurane inhalation maintained stable BP, however, continuous surgical plain of anesthesia was achieved only with isoflurane inhaled at 1%. Thus isoflurane 1% inhalation anesthesia is recommended to be used for experiments requiring prolong stable BP in anesthetized rats.

**604**

**Effects of fluoride on the tissue oxidative stress and apoptosis in rats**

Chouhan S, Flora S J S

**Defence Research & Development Establishment, Gwalior, India.**

The aim of the present study has been to determine the effect of fluoride on tissue oxidative stress and apoptosis exercised by the administration of different doses of fluoride. Thirty male rats were exposed to three doses of fluoride (10 ppm, 50ppm and 100 ppm in drinking water) for a period of 10 weeks. The results suggested that exposure to 10 mg/l fluoride significantly increased the level of Reactive Oxygen Species (ROS) in blood accompanied by a decrease in glutathione (GSH) level. No evidences of oxidative stress in soft tissues were seen. Fluoride (10 mg/l) also decreased GSH/ GSSG ratio significantly. Contrary to expectation, 50 and 100 mg/l fluoride exposure did not produce a more pronounced toxicity in the soft tissues. However, we observed a significantly elevated concentration of ROS and depleted GSH level in blood. Exposure to fluoride did not produce any sign of apoptosis. To support our above mentioned biochemical observations and to suggest possible mechanism of action of fluoride, IR spectra of brain tissues were recorded. The results of these spectra indicated significant shift in the characteristic peak of –OH group in animals exposed to 10 mg/l fluoride however at higher doses, the shift was minimal. It can thus be concluded that fluoride induced toxicity is mediated through oxidative stress particularly at a comparatively lower level of exposure however at the higher doses the mode of action still unclear and needs further investigation.

**605**

**Mitigation of metformin-induced thyroid dysfunctions by *Withania somnifera* extract in type 2 diabetic mice**

Jatwa R, Kar A

**Devi Ahilya University, Indore, India.**

The present study was carried out to reveal the possible ameliorative role of a plant extract on anti-diabetic drug-induced thyroid dysfunctions in an animal model of type 2 diabetes mellitus. Administration of dexamethasone (1.0 mg / kg, i.m.) caused hyperglycemia with a parallel increase in the concentrations of serum insulin, total cholesterol (TC), low-density lipoprotein cholesterol (LDL-C), very low-density lipoprotein cholesterol (VLDL-C) and triglycerides (TG) as well as in renal lipid peroxidation (LPO) and relative risk ratio (RR). It decreased serum triiodothyronine (T3), thyroxine (T4) and high-density lipoprotein cholesterol (HDL-C) levels as well as renal superoxide dismutase (SOD), catalase (CAT) and reduced glutathione (GSH) content. Administration with metformin (150 mg / kg, p.o.), a widely used oral hypoglycemic agent, to diabetic animals further reduced circulating thyroid hormones and caused drug-induced hypothyroidism. It also reduced renal LPO and RR value; serum concentrations of insulin, glucose and LDL-C with a parallel increase in endogenous antioxidants. While oral administration with Withania somnifera (1.4 g / kg) root extract along with dexamethasone and metformin elevated the concentrations of circulating T3 and T4 to euthyroid level. The plant extract also corrected RR ratio and serum concentration of lipids. These findings, for the first time, reveal that the evaluated plant extract has a potential to ameliorate metformininduced hypothyroidism in type 2 diabetic subjects.

**606**

**A comparative study of antihypertensive drugs in isolated systolic hypertension**

Revathy saravanan^1^, C B Tharani, B Krishnaswamy

**Chettinad Medical College, Chennai**

**Objectives:**

To compare the efficacy of available antihypertensive drugs in I.S.H. and to asses the tolerability& Pharmaco economical analysis of the drugs.

**Methods:**

Study centre: Geriatric O.P. Govt. General Hospital Chennai. Study duration:--6 months. Study design: Prospective Single Blind nonrandomized comparative study. New I.S.H patients of both sexes who fulfilled the criteria were included & allotted to five groups in nonrandomized way after taking informed consent. I.S.H has been defined as S.B.P of 140mm of Hg or more and D.B.P 90mms or < 90mms of Hg.

Group A: Hydrochlorthiazide-12.5-25mg a day
Group B: Atenolol-50-100 mg a day
Group C: Nifedipine 10-30mg a day as single or divided doses
Group D: Amlodipine 2.5-7.5mg once a day
Group E Enalapril 2.5-10 mg a day as single or divided dose

Sitting B.P. recordings were tabulated and analyzed using ANOVA and bonferroni multiple comparison “t” test.

**Results:**

No of I.S.H patients-67 (10.8% of hypertensive and 3.3% of O.P cases). 61 patients completed study. Males (59%) were more than Females (41%) and 2 patients did not respond in thiazide group. All the drugs showed marked reduction in systolic B.P compared to basal visit with p value of 0.001 with no difference between groups. Maximum reduction of 36 mms of Hg was seen with ATENOLOL and minimum of 17mms of Hg with thiazide. All the groups showed gradual and minimal reduction in D.B.P.

**Conclusion:**

Amlodipine 2.5-5 mg once a day at night is preferred in I.S.H.

**607**

**Prescribing practices of psychiatrists in treatment of depression**

Maduram A, Parvathavarthini S

**Chettinad Medical College, Chennai**

**Introduction:**

The past decade has seen an increase in the number and type of antidepressants available to psychiatrists and other clinicians. In Chennai, Madurai and Pondicherry of India there are at least 13 antidepressants available. It is important to better understand current prescribing practices and to what degree these practices reflect research findings.

**Objectives:**

To examine the current prescribing practices in government and private clinics in Chennai, Madurai and Pondicherry. To find out the choice of antidepressant drugs by psychiatrists. To determine the most commonly prescribed daily dose of antidepressant drugs. To evaluate the antidepressant group causing least or most side effect weight gain, agitation, sexual dysfunction, insomnia and discontinuation of depression therapy. To find out the factors influencing choice of antidepressant in the treatment of depression.

**Methods:**

A cross – sectional study. Center – Tamil Nadu and Pondichery. Duration 5 months Self administrated questionnaire was asked to be filled. Overall response rate of 97%. luoxetine and Citalopram was the most are favored as first line treatment in a first episode of depression and are prescribed more frequently than TCA agents.

**Results:**

The meta-analyses showed that SSRIs has greatest efficacy, least side effects, causing sexual dysfunction, discontinuation of treatment, causing agitation and insomnia. Doctors in the psychiatric services were prescribing recommended dosages of antidepressants for an adequate time period.

**Conclusion:**

SSRIs were deemed to be the class of drugs that would most likely be prescribed.

**608**

**Oral salt load tolerability and its effect on arterial blood pressure of conscious SHRsp implanted with radiotelemetry transmitter**

Mathur A, Gupta R, Mohanan A, Joshi D

**Torrent Pharmaceuticals Ltd., Research Centre, Gandhinagar, India.**

It has been estimated that approximately one half of all hypertensive patients have some degree of salt sensitivity, defined as 10mmHg drop in blood pressure (BP) when consuming a low salt versus a high salt diet. The present study was designed to establish tolerability of SHRsp to various salt concentrations and determine whether hypertension observed in SHRsp is sensitive to salt load or not.

SHRsp rats were implanted with radiotelemetry transmitters. Under isoflurane anesthesia, gel-filled telemetry catheter was introduced into the descending aorta above the iliac bifurcation and pushed just caudal to the renal arteries. The catheter was secured at the entry to the vessel and the transmitter body was sutured to the inner peritoneal wall before the midline incision was closed. After recovery from surgery, rats were provided with 1% saline for 13 days, followed by 2% saline for next 6 days and eventually to 4% saline for a week. Throughout the study, BP was recorded with help of computer driven data acquisition system.

Telemetry enabled us to record BP round the clock from unrestrained, conscious SHRsp. A dose dependent increase in BP of SHRsp was observed with increase in salt load. SHRsp tolerated gradual increment of salt from 1% to 2%, however, failed to tolerate 4% salt in drinking water, as was evident from clinical observations and died eventually while on 4% salt within a week. This method validated the salt loaded telemetered model for recapitulating the pathophysiology of salt sensitive hypertension in the clinical situation.

**609**

**A questionnaire based survey of self medication behavior among non-medical and medical students in india**

Kumar R, Padhy B M, Reeta K H, Gupta Y K

**Department Of Pharmacology, All India Institute Of Medical Science, New Delhi, India.**

**Objectives:**

To assess the pattern of self-medication behavior prevalent among non-medical and medical students.

**Method:**

A questionnaire based cross-sectional survey was conducted on 338 students (238 medical and 100 non-medical students).

**Results:**

A total of 225 female and 113 male students in the age group of 18-25years (mean age 22.5 years) from 27 colleges participated in the study. Among medical students, 62% were MBBS students, 27% nursing students, 10% paramedical students and 1% BDS student. Among non-medical students, 67% were graduate students and 33% postgraduate students. Parents of 94% students were unrelated to health profession while 6% were related to health profession. Among non-medical students, the most commonly used medications without valid prescription were antibacterial (54%), antitussives (52%), analgesics (39%), antipyretics (34%), and homeopathic medicines (22%). Among the medical students, out of 16 categories of drugs, the most commonly used medications without valid prescription were analgesics (63%), antipyretics (50%), antibacterial (40%), and antacids (35%). There was no authentic source to guide the correct dose. Most drugs were taken for symptomatic relief. 81% of them got the medicine from market while 19% from physician’s sample or medical representatives. Majority of students were not aware about the cost of medicine. Among the 238 medical students, (86%) had knowledge about adverse effects of medicine, while only (44%) non-medical students were aware of the adverse effects of medicine.

**Conclusion:**

This study indicated that self medication is common among non-medical and medical students in India. Creating awareness about the rationale use of drugs and strict regulatory control on availability of drugs may reduce the irresponsible self medication behavior.

**610**

**Diuretic activity of Raphanus sativus**

K Hari Kumar, M Charles, Ravi kumar, M Vasanta

**Department of Pharmacy, Sri Venkateswara College of Biological and Earth Sciences, S.V. University, Tirupati. Andhra Pradesh**

According to folklore medicine the leaves of Raphanus sativus have known to show diuretic activity. Lipschitz method using albino rats assessed the diuretic activity of raphanus sativus. The aqueous and alcoholic extracts of the leaves of raphanus sativus were extracted for 15 days using a soxhlet apparatus.. Experimental albino rats (both male and female) were divided into four groups, control group (saline), test group 1(saline+alcoholic extract), test group2 (saline+aqueous extract), standardgroup (saline+furosemide) each group containing 15 animals. All the parameters of animals like body weight, bmi (body mass index), sodium and potassium content in urine, volume of urine were studied. Ed50 and ld50 values for all the animals was assessed and the therapeutic index of the drug was determined. Water and sodium excretion in test animals was compared to the rats treated with high dose of creatinine 4ml/100g body weight along with drug was administered to all the animals through intraperitonial route. The animals were placed in metabolic cages overnight and the amount of urine excreted was recorded. The animals were made to go through the same routine everyday for 90 days. The data was collected and analyzed using statistical methods. Test group2 has shown an increase in urine output when compared test1 and control groups. In conclusion it can be deduced that the greater diuretic activity in the test2 group when compared to other groups can be attributed to the chemical constituents that get dissolved in the aqueous extract. The higher diuretic activity can be attributed to the minerals like mercury (hg), potassium (k), sodium (na), carbohydrates like glucoside and sucrose and vitamins. These are known to act by increasing the tonicity inside the renal tubules and thereby causing osmotic diuresis.

**611**

**A study on prescribing trends and rational use of drugs in an urban area of jaipur**

Mathur P^1^, Mathur M^2^, Sinha M^3^

**^1^Apex Institute of Mangement and Science, Jaipur, India, ^2^SMSMedical College, Jaipur, India, ^3^Sri Balaji College of Pharmacy, Jaipur, India.**

**Introduction:**

Appropriate use of drugs is of critical importance for achieving good health and efficient use of resources in healthcare system. The proper use of medicines is relevant for patients, healthcare professionals and regulators. In view of its importance, a survey was conducted to assess the prescribing practice in outpatients in Jaipur.

**Methods:**

The prospective study involved review of prescription.s data. Community pharmacists practicing in high population density area in Jaipur were approached to provide photocopies of the prescriptions dispensed. A total of 99 prescriptions, written by doctors of differing specialties, were randomly collected from 5 different retail pharmacies in Jaipur and analyzed.

**Results:**

A total number of 364 medicines (mean per prescription 3.67; range 1 to 9) were recorded. About 67% of the patients were prescribed formulations containing drug combinations. A total of 93 combinations were prescribed. The five most common combinations were of NSAIDS, vitaminsminerals- enzymes, antibiotics, prebiotic-probiotic & antidiabetic drugs. The data also revealed a high incidence of antimicrobials use. A total 64 prescriptions contained 75 antimicrobials or their combinations out of which 10 prescriptions had two or more antimicrobials. Most commonly used antimicrobials were amoxicillin-clavunate combination followed by cephalosporins.

**Conclusion:**

It is concluded that despite the emphasis on rational prescribing & discouraging injudicious use of antimicrobials and drug combinations, such prescriptions are still common. It is necessary to persist in sensitizing the healthcare professionals on the principles of rational prescribing.

